# Decreased mortality and increased side effects in COVID-19 patients treated with IL-6 receptor antagonists: systematic review and meta-analysis

**DOI:** 10.1038/s41598-021-00726-4

**Published:** 2021-11-02

**Authors:** Jishnu Malgie, Jan W. Schoones, Maurice P. Zeegers, Bart G. Pijls

**Affiliations:** 1 Department of Orthopaedics, Leiden University Medical Center, Albinusdreef 2, Postzone J-11-S, P.O. Box 9600, 2300 RC Leiden, The Netherlands; 2 Directorate of Research Policy (Formerly: Walaeus Library), Leiden University Medical Centre, Leiden, The Netherlands; 3 Team Meta-Research, NUTRIM School of Translational Research in Metabolism, CAPHRI, Care and Public Health Research Institute, Maastricht University, Maastricht, The Netherlands

**Keywords:** Immunotherapy, Infectious diseases, Viral infection, Epidemiology

## Abstract

There is controversy whether IL-6 (receptor) antagonists are beneficial in treating COVID-19 patients. We therefore update our systematic review to answer the following research questions: (1) Do patients hospitalized for COVID-19 treated with IL-6 (receptor) antagonists have lower mortality compared to standard of care? (2) Do patients hospitalized for COVID-19 treated with IL-6 (receptor) antagonists have more side effects compared to standard of care? The following databases were search up to December 1st 2020: PubMed, PMC PubMed Central, MEDLINE, WHO COVID-19 Database, Embase, Web-of-Science, COCHRANE LIBRARY, Emcare and Academic Search Premier. In order to pool the risk ratio (RR) and risk difference of individual studies we used random effects meta-analysis. The search strategy retrieved 2975 unique titles of which 71 studies (9 RCTs and 62 observational) studies comprising 29,495 patients were included. Mortality (RR 0.75) and mechanical ventilation (RR 0.78) were lower and the risk of neutropenia (RR 7.3), impaired liver function (RR 1.67) and secondary infections (RR 1.26) were higher for patients treated with IL-6 (receptor) antagonists compared to patients not treated with treated with IL-6 (receptor) antagonists. Our results showed that IL-6 (receptor) antagonists are effective in reducing mortality in COVID-19 patients, while the risk of side effects was higher. The baseline risk of mortality was an important effect modifier: IL-6 (receptor) antagonists were effective when the baseline mortality risk was high (e.g. ICU setting), while they could be harmful when the baseline mortality risk was low.

## Introduction

Immunomodulatory agents such as corticosteroids have been shown to decrease mortality in COVID-19 patients^1^. For a more targeted approach IL-6 (receptor) antagonist have been suggested as an effective treatment for severe COVID-19. Our previous rapid systematic review on observational studies suggested a benefit for IL-6 (receptor) antagonist on mortality for COVID-19 patients^2^. However, the results from early RCTs seem to contradict these findings^3^. Since the results from several randomized trials have now been published, an update of the review could provide new insights^4^.

We therefore updated our systematic review to answer the following research questions: (1) Do patients hospitalized for COVID-19 treated with IL-6 (receptor) antagonists have lower mortality compared to standard of care? (2) Do patients hospitalized for COVID-19 treated with IL-6 (receptor) antagonists have more side effects compared to standard of care?

## Methods

The reporting of this meta-analysis is in accordance with the PRISMA-statement^5^. The methods and early results have been published previously^2^. We set out to search the literature on studies comparing either IL-6 receptor antagonists (tocilizumab, sarilumab), or IL-6 antagonists (siltuximab) to a control group in COVID-19 patients. Both non-randomised intervention studies and randomized controlled trials were considered. The primary outcome was mortality, expressed as the number of patients who died within the study period. The secondary outcomes were mortality in ICU, risk of mechanical ventilation, composite of mortality and mechanical ventilation, and possible side effects of Il-6 (receptor) antagonists such as secondary infection, neutropenia, intestinal perforation and impaired liver function.

### Data sources and searches

The search strategy was composed in collaboration with a librarian (JS). The following databases were searched from their inception up to 1 December 2020: PubMed, PMC PubMed Central, MEDLINE, WHO COVID-19 Database, Embase, Web of Science, COCHRANE LIBRARY, Emcare and Academic Search Premier. The search strategy consisted of the following components, each defined by a combination of controlled vocabulary and free text terms:

1. Anti-IL-6 treatment
2. COVID-19

The full search strategy is provided in the Online Appendix.

### Study selection

Studies identified by the search strategy were screened on title and abstract. This screening was performed by two reviewers (JM and BP) independently. Both reviewers recorded their findings in a pre-designed electronic database. Both databases were then compared and any disagreements were resolved by consensus. When the information in the abstract did not suffice, or if any doubt remained, the studies remained eligible.

The full text articles of eligible studies were independently evaluated by two reviewers (JM and BP). Both recorded their findings in a pre-designed electronic database. Any disagreements were resolved by consensus.

All bibliographic records identified through the electronic searches were collected in an electronic reference database and subjected to the following inclusion and exclusion criteria:

Inclusion criteria:

1. Adult COVID-19 clinical patient study (both observational studies and randomized controlled trials were considered)
2. Anti-IL-6 therapy versus non-anti-IL-6 therapy with a minimum of 5 patients in each treatment arm

Exclusion criteria:

1. No clinical study on hospitalized COVID-19 patients
2. No intervention (IL-6 therapy) versus control (standard of care)
3. No data on primary and secondary outcomes
4. anti-IL-6 therapy reserved for severe or cytokine storm patients (severe and apparent confounding by indication), while mild patients get standard therapy
5. Language not spoken by review team

### Data extraction and quality assessment

Two reviewers (JM and BP) independently extracted data and appraised the risk of bias from included studies regarding the primary and secondary outcomes, patient demographics and study characteristics in a pre-defined electronic data sheet: author, journal, country, study type, clinical setting, concomitant medication (e.g. glucocorticosteroids, azithromycin), type of anti-IL-6 medication, anti-IL-6 dose and administration route, number of patients who received anti-IL-6 treatment or control, number of patients who reached primary and secondary outcomes, crude and adjusted estimates for primary outcome including standard errors and confidence intervals, mean age of patients, number of men/women, mean IL-6 levels at baseline, mean CRP levels at baseline, number of patient with diabetes mellitus, hypertension and cardiovascular diseases, mean BMI, mean PaO2:FiO2 ratio at baseline, mean duration of symptoms, funding and potential conflict of interests. The data sheet was designed during the extraction of trial data on a random sample of eligible studies. Any disagreements were resolved by consensus.

Risk of bias was appraised using the MINORS checklist for observational studies and the Cochrane Risk of Bias tool 2 (RoB2) for RCTs^6,7^. MINORS is specifically designed to assess the methodological quality of non-randomized studies^6^. A MINORS item scored 0 if not reported, 1 if reported but not adequate and 2 if reported and adequate. With 12 items this gives a maximal possible score of 24 points. We considered a study of high quality if the total MINORS score was 20 or more and low quality if the total score was less than 20^2^. To assess the certainty of the evidence, we followed the Grading of Recommendations Assessment, Development and Evaluation (GRADE) recommendation^8^.

### Data synthesis and analysis

For the meta-analysis we used a random effects model to pool the risk ratio and risk difference of individual studies in order to estimate an overall risk ratio and risk difference (absolute risk difference) along with their associated confidence intervals for each of the outcome measures^9^. The risk difference was included, because it it allows calculation of the number needed to treat (NNT)^10,11^. The amount of statistical heterogeneity was assessed through visual inspection of the forest plots and by calculating I^2^ statistics^12^. The I^2^ statistic estimates how much of the total variability in the effect size estimates is due to heterogeneity among the true effects. In the presence of heterogeneity, and if the data allowed, we performed a random effects meta-regression on pre-defined factors (study level covariates). All analyses were performed using Metafor Package R statistics^13^.

We constructed a funnel plot for studies reporting the primary outcome to assess the amount of publication bias. In case the funnel plot was asymmetric, we used a trim-and-fill to explore the magnitude and direction of the publication bias.

## Results

### Study selection and study characteristics

**Figure 1:**
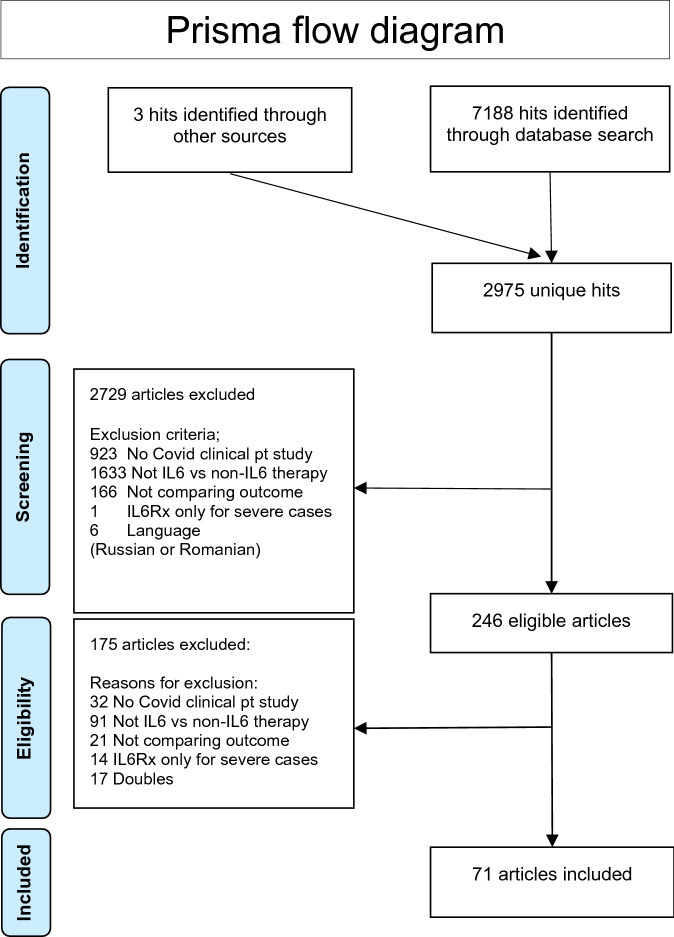
Prisma flow chart^5^.

The search strategy retrieved 7191 hits of which 2975 were unique (no double entries for different databases). After selection 71 studies were included with a total of 8652 patients who received Il-6 (receptor) antagonists and 20,843 patients in the control group who did not receive Il-6 (receptor) antagonists. In 69 studies tocilizumab was used, in 2 studies sarilumab and in 1 study siltuximab. Details of the study selection and the flowchart of the review are shown in Fig. 1.

From the included studies 24 were from the USA, 16 from Italy, 12 from Spain, 7 from France, 2 from Brazil, 2 from India and one from each of the following countries: China, Russia, Sweden and the UK. Additionally, 3 studies included patients from around the world and one study included European patients.

From the included studies 9 were RCTs and 62 were observational studies. Regarding the clinical setting, 57 studies included hospitalized patients, while 14 studies (including 1 RCT with both tocilizumab and sarilumab compared to controls) were restricted to ICU patients.

### Risk of bias

Details on the risk of bias are presented in the Online Appendix. For observational studies, the mean MINORS score was 19.4 (range 13–22) out of 24 points. 35 studies were considered to be of high quality with a MINORS score of 20 or higher and 27 studies were considered of lower quality. For the RCTs, the overall risk of bias as assessed by ROB2 suggested low risk of bias to some concerns, see Online Appendix, which were mostly related to open label (unblinded) design, differences in co-medications such as steroids, differences at baseline regarding age and inflammatory markers and in one RCT TCZ was used as rescue medication in 14 of 66 patients^14^.

### Synthesis of the results and sensitivity analyses

#### Primary outcome

**Figure 2:**
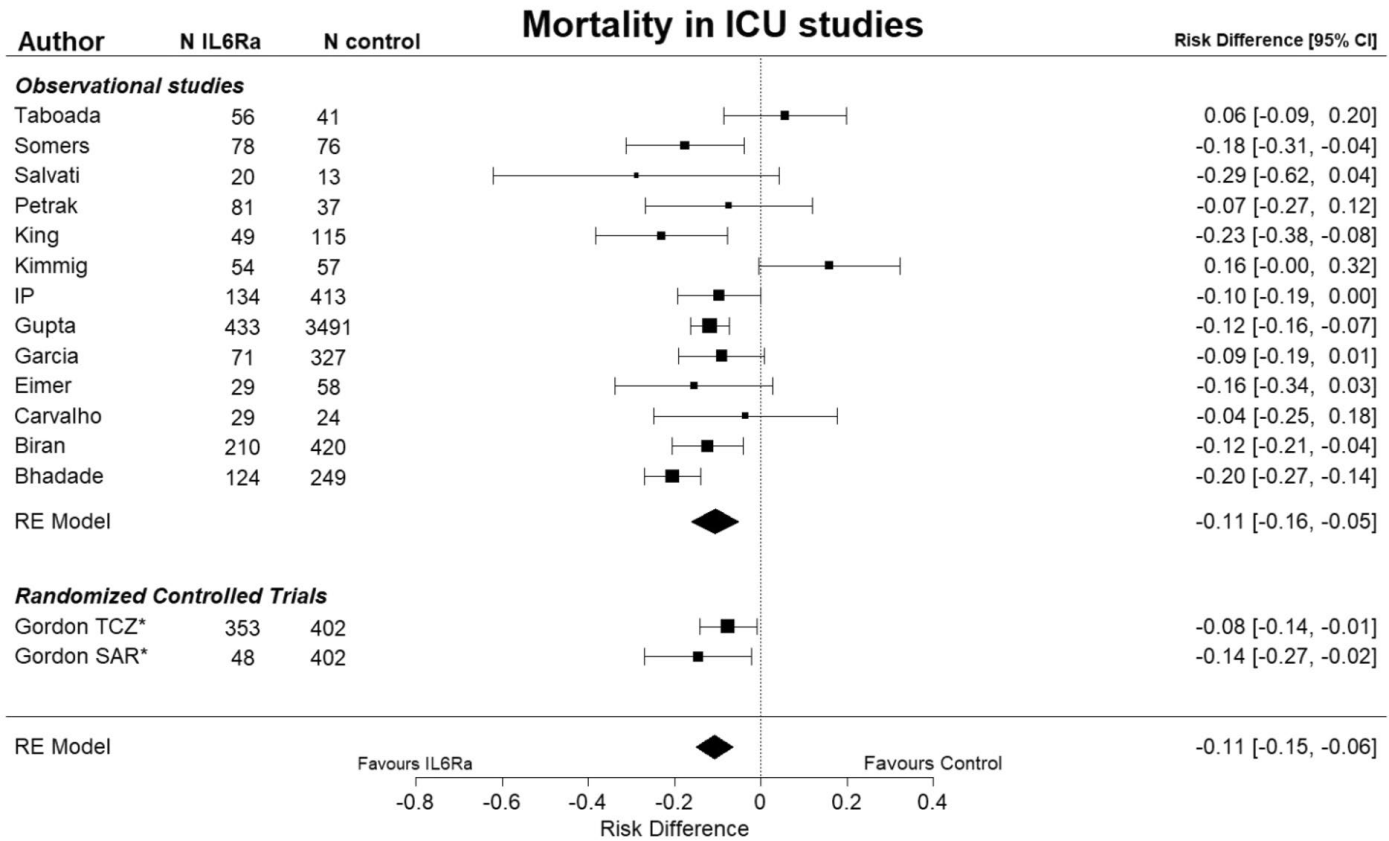
Forest plot showing the risk difference for studies in ICU setting in mortality between patients treated with IL-6 (receptor) antagonists and patients not treated with IL-6 (receptor) antagonists. I^2^ was 0%. The Metafor R package was used to generate this figure^13,23^. IL6Ra = IL-6 (receptor) antagonist, TCZ = tocilizumab, SAR = sarilumab, * = RCT.

**Figure 3:**
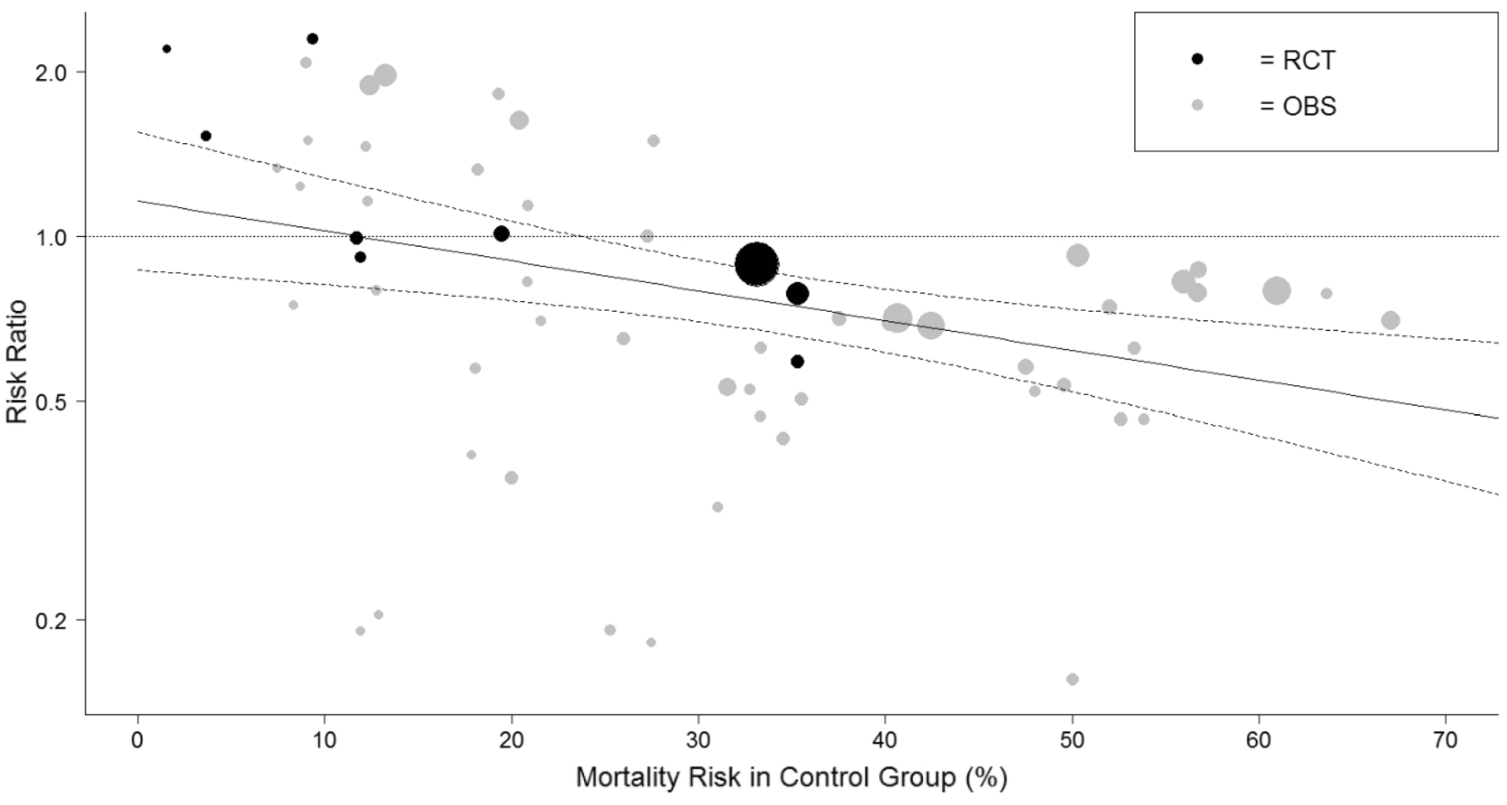
Graph showing the results from the meta-regression on baseline mortality risk, defined as mortality risk in the control group, and relative risk of mortality for patients treated with IL-6 (receptor) antagonists and patients not treated with IL-6 (receptor) antagonists. The size of the circles is inversely proportional to the variance of the estimated treatment effect. The Metafor R package was used to generate this figure^13,23^. The dashed lines represent the limits of the 95% confidence interval. RCT = randomized controlled trial (black dots). OBS = observational study (gray dots).

Table 1 shows a summary of the data-synthesis. Based on 63 studies with 23,826 patients mortality was lower in the IL-6 group compared to the control group with a risk ratio (RR) of 0.75 (95% CI 0.65 to 0.86) and a risk difference (RD) of 7.4% (95% CI 4.4% to 10%), see Fig. S1. A sensitivity analysis was necessary because the heterogeneity was substantial, I^2^ was 82%. There was no remaining heterogeneity (I^2^ was 0%) when the meta-analysis was restricted to studies in the ICU setting, while the mortality remained lower for the IL-6 group compared to the control group: based on 15 studies with 7894 patients the RR was 0.75 (95% CI 0.62 to 0.96) and the RD was 11% (95% CI 6.4% to 15%), see Fig. 2. These results suggest that the baseline risk of mortality could be an effect modifier, so a meta-regression was performed on the RR of mortality versus the risk of mortality in the control group. Figure 3 shows the results of this meta-regression: the RR of mortality deceases when the risk in the control group increases and a beneficial effect of IL-6 (receptor) antagonists on reducing mortality was seen when the mortality risk in the control group was 25% or more. Baseline risk of mortality remained an effect modifier when the analyses was restricted to RCTs (*p* = 0.033).

**Table 1:** summary of data synthesis.

	Outcome	Number of Il-6 study groups	Number of patients	Pooled estimate (RR) [95% CI]	Pooled estimate (RD) in % [95% CI]	Heterogeneity (I^2^)	GRADE
Treatment outcome	Mortality *all studies*	63	23,826	0.75 [0.65 to 0.86]	− 7.4 [− 10 to − 4.4]	82%	
	Mortality *RCTs only*	9	6765	0.88 [0.82–0.96]	− 1.3 [− 4.4 to − 1.9]	0%	M–H
	Mortality *all studies* only ICU studies	15	7894	0.75 [0.70–0.82]	− 11 [− 15 to − 6.4]	0%	
	Mortality *RCTs only* ICU studies	2	803	0.76 [0.62 to 0.94]	− 8.4 [− 15 to − 2.0]	0%	M–H
	Mechanical Ventilation *all studies*	23	3165	0.78 [0.62–0.96]	− 6.9 [− 11 to − 2.3]	71%	
	Mechanical Ventilation *RCTs only*	4	941	0.68 [0.50–0.92]	− 5.9 [− 10 to − 1.3]	0%	M–H
	ICU admission *all studies*	15	4866	1.3 [0.79 to 2.1]	3.2 [− 6.4–13]	91%	
	ICU admission *RCT only*	3	695	0.60 [0.42–0.87]	− 6.1 [− 15 to − 3.0]	0%	M
	Composite of mortality and mechanical ventilation *all studies*	12	7122	0.66 [0.54–0.82]	− 10 [− 16 to − 4.8]	47%	
	Composite of mortality and mechanical ventilation *RCTs only*	6	5008	0.85 [0.78–0.93]	− 4.6 [− 7.0 to − 2.1]	0%	M–H
Side effect	Secondary infection *all studies*	39	15,345	1.11 [0.90–1.37]	1.3 [− 1.4–3.7]	86%	
	Secondary infection *RCTs only*	8	2649	0.73 [0.56–0.96]	− 2.1 [− 4.7–0.6]	0%	M
	Neutropenia *all studies*	9	1427	7.3 [2.6–20]	7.6 [1.3–14]	0%	
	Neutropenia *RCTs only*	3	498	7.7 [1.8–33]	6.1 [− 0.3–13)	0%	M–H
	Impaired liver function *all studies*	14	6444	1.67 [1.4–2.0]	6.3 [0.01–12]	0%	
	Impaired liver function *RCTs only*	3	810	1.25 [0.6–2.6]	0.2 [− 2.2–2.6]	0%	M–H
	Pulmonary Embolism *all studies*	7	4631	1.2 [0.72–2.1]	0.0 [− 3–3]	38%	
	Pulmonary Embolism *all studies*	2	372	0.3 [0.07–1.06]	− 2.2 [− 5.4–1.0]	0%	M

*RR* risk ratio; defined as: risk tocilizumab group/risk control group, *RD* risk difference; defined as: risk tocilizumab group—risk control group. *95% CI* 95% confidence interval, *M* moderate certainty, *M–H* moderate to high certainty.

In observational studies (n = 54) the IL-6 group had lower mortality than the control group: the RR was 0.71 (95% CI 0.61 to 0.83) and the RD was 8.8% (95% CI 5.4% to 12%) with considerable remaining heterogeneity I^2^ of 83%. In RCTs (n = 9) the IL-6 group had lower mortality than the control group: the RR was 0.88 (95% CI 0.82 to 0.96) and the RD was 1.3% (95% CI − 1.9% to 4.4%) with no remaining heterogeneity I^2^ of 0%. The overall mortality in the control groups was approximately 18% (95% CI 9% to 27%) for the RCTs compared to 31% (95% CI 27% to 36% ) for the observational studies. Therefore we performed a sensitivity analysis restricting observational studies to studies with mortality rates in the control group of 35% or less, which is to the highest control group mortality of the RCTs (*p* = 0.37). In this sensitivity analysis the results of the observational studies were similar to those of the RCTs: RR was 0.79 (95% CI 0.61 to 1.02) and the RD was 3.8% (95% CI − 0.4% to 7.8%) for the observational studies and the RR was 0.88 (95% CI 0.82 to 0.96) and the RD was 1.3% (95% CI − 1.9% to 4.4%) for the RCTs.

A trim and fill analysis was warranted, because the funnel plot showed some asymmetry on visual inspection (Egger test *p* = 0.059; Begg test *p* = 0.55). The trim and fill analysis showed small influence of possible publication bias. The RR was 0.74 (95% CI 0.65 to 0.86) compared to 0.75 and the RD was 4.3% (95% CI 1.1% to 7.6%) compared to 7.4%. When restricting the analyses to studies with a MINORS score of 20 points or more (high quality) and RCTs: the RD was 9.1% (95% CI 5.6% to 13%) in favour of the IL-6 group and the RR was 0.69 (95% CI 0.59 to 0.80) in favour of IL-6 group based on 40 studies. There were 36 studies that presented adjusted analyses to correct for differences at baseline. These analyses confirmed the lower mortality for the IL-6 group: adjusted RR was 0.56 (95% CI 0.45 to 0.72) with I^2^ of 87%.

When restricted to tocilizumab, the mortality was lower in the tocilizumab group compared to the control group based on 60 studies: the RR was 0.76 (95% CI 0.66 to 0.87) and the RD was 7.2% (95% CI 4.1% to 10%), with substantial heterogeneity I^2^ of 82%. When the meta-analysis was restricted to studies conducted in the ICU setting there was no remaining heterogeneity (I^2^ of 0%), while the mortality in the tocilizumab group remained lower than in the control group: based on 14 studies with 7444 patients the RR was 0.76 (95% CI 0.70 to 0.82) and the RD was 10% (95% CI 5.7% to 15%).

The effect of IL-6 (receptor) antagonists on mortality was smaller in studies that used glucocorticoids (RR was 0.80 (95% CI 0.70 to 0.90)), compared to studies in which no glucocorticoids were used (RR was 0.33 (95% CI 0.19 to 0.60)). The effect of IL-6 (receptor) antagonists on mortality was smaller in studies that used azithromycin (RR was 0.79 (95% CI 0.65 to 0.94)), compared to studies in which no azithromycin was used (RR of 0.36 (95% CI 0.18 to 0.71)).

Secondary outcomes: a summary of the data-synthesis is presented in Table 1 and Figs. S2 through S8. The risk of mechanical ventilation (23 studies) and the composite outcome of mortality and mechanical ventilation (12 studies) were lower for patients treated with Il-6 (receptor) antagonists compared to patients not treated with IL-6 (receptor) antagonists: RR 0.78 (95% CI 0.62–0.96) for mechanical ventilation and RR of 0.66 (95% CI 0.54 to 0.82) for the composite endpoint see Figs. S2 and S4. There was no difference observed in the risk of ICU admission (15 studies): RR 1.3 (95% CI 0.79 to 2.1) see Fig. S3. There was a higher risk of neutropenia (9 studies) for patients treated with Il-6 (receptor) antagonists compared to patients not treated with IL-6 (receptor) antagonists: RR 7.3 (95% CI 2.6 to 20) see Fig. S6. There was a higher risk of impaired liver function (14 studies) for patients treated with Il-6 (receptor) antagonists compared to patients not treated with IL-6 (receptor) antagonists: RR 1.67 (95% CI 1.4 to 2.0) see Fig. S7. There was no difference observed in the risk of pulmonary embolism (7 studies): RR 1.2 (95% CI 0.72 to 2.1) see Fig. S8. There was no difference observed in the risk of secondary infections (39 studies): RR 1.1 (95% CI 0.90 to 1.37) with considerable heterogeneity I^2^ of 86% see Fig. S5. When the meta-analysis was restricted to studies conducted in the ICU setting (9 studies) the risk for secondary infections was higher for patients treated with Il-6 (receptor) antagonists compared to patients not treated with IL-6 (receptor) antagonists: RR 1.26 (95% CI 1.08 to 1.82) I^2^ of 52%.

## Discussion

### Summary of evidence

In this systematic review and meta-analysis we re-evaluated the treatment effect of IL-6 (receptor) antagonists on mortality and possible side effects in COVID-19 patients compared to COVID-19 patients who did not receive IL-6 (receptor) antagonists^2^. Our results showed that IL-6 (receptor) antagonists were associated with a reduction in mortality, a reduction in mechanical ventilation and a reduction in the composite endpoint of mortality and mechanical ventilation for COVID-19 patients based on moderate to high certainty evidence. We observed no effect of IL-6 (receptor) antagonists on the risk of ICU admission. However, there was considerable heterogeneity of 91% meaning that there was substantial variation of the results between studies.

Regarding possible side effects, our results showed that IL-6 (receptor) antagonists were associated with an increased risk of neutropenia and an increased risk of impaired liver function based on moderate to high certainty evidence. We observed no effect of IL-6 (receptor) antagonists on the risk of pulmonary embolism or the risk of secondary infections based on moderate certainty evidence. However, when the meta-analysis was restricted to studies conducted in the ICU setting (9 studies) the risk for secondary infections was higher for patients treated with Il-6 (receptor) antagonists compared to patients not treated with IL-6 (receptor) antagonists.

Careful patient selection and timing for IL-6 (receptor) antagonist treatment in COVID-19 patients is crucial^15–17^, especially considering the increased risk of side effects—neutropenia, impaired liver function and secondary infections—when IL-6 (receptor) antagonists were used. Our analyses suggested that Il-6 (receptor) antagonist treatment is beneficial in reducing mortality for patients with a high baseline risk of mortality, while it may be harmful for patients with a low baseline risk of mortality.

### Limitations and strengths

We should consider some limitations. The fact that we used crude risks for the calculation of RR and RD can be considered a limitation, as this does not allow control of baseline imbalances by treatment group. However, 36 studies reported adjusted analyses to account for differences at baseline. These analyses confirmed the lower mortality for the IL-6 group: adjusted RR was 0.56 (95% CI 0.45 to 0.72).

The fact that we included observational studies and did not restrict our systematic review to RCTs may be considered a limitation. However, this allowed us to explore possible differences between the results from observational studies and RCTs^18–20^. Our analyses suggested that the patients in observational studies were different from patients in RCTs in terms of their baseline risk of mortality: the overall mortality in the control groups was approximately 18% for the RCTs, which was much lower than the mortality of 31% for the observational studies. Therefore we performed a sensitivity analysis which was restricted to observational studies with similar mortality rates in the control group as the RTCs (i.e. 35% or less) to reflect more similar patient populations. In this sensitivity analysis the results of the observational studies were not apparently different to those of the RCTs: RR was 0.79 (95% CI 0.61 to 1.02) for the observational studies compared to RR of 0.88 (95% CI 0.82 to 0.96) for the RCTs. These results are in line with other systematic reviews which have shown that results from observational studies are not apparently different from results of RCTs^18–21^. Furthermore the results from the WHO Rapid Evidence Appraisal for COVID-19 Therapies (REACT) Working Group, meta-analysis on only RCTs, confirmed our conclusion that IL-6 (receptor) antagonists are associated with lower mortality for patients with COVID-19^22^.

The strengths of the review are: two reviewers performed all phases of the review in duplo and the conclusions did not change after meticulous sensitivity analyses. The results also remained similar when the analyses were restricted to RCTs and observational studies of a MINORS score of 20 reflecting the highest methodological quality. Funnel plots and trim-and-fill analyses suggested that the influence of publication bias was negligible. Additionally, we were able to identify several factors that were considered effect modifiers on the efficacy of IL-6 (receptor) antagonists in COVID-19 patients: clinical setting (ICU), baseline risk of mortality, use of glucocorticoids and use of azithromycin.

## Conclusion

Meta-analyses on 71 studies comprising 29,495 patients showed that mortality, mechanical ventilation and the composite endpoint of mechanical ventilation and mortality were lower for COVID-19 patients treated with IL-6 (receptor) antagonists compared to COVID-19 patients who were not treated with IL-6 (receptor) antagonists, whereas the risk of neutropenia, impaired liver function and secondary infections were higher for patients treated with IL-6 (receptor) antagonists compared to patients not treated with treated with IL-6 (receptor) antagonists. The baseline risk of mortality was an important effect modifier: IL-6 (receptor) antagonists were effective when the baseline mortality risk was high (e.g. ICU setting), while they could be harmful when the baseline mortality risk was low.

## Supplementary Information

Supplementary Information.
