# Supplementary material for: Decreased mortality and increased side effects in COVID-19 patients treated with IL-6 receptor antagonists: systematic review and meta-analysis
Source: Sci Rep. 2021 Nov 2;11:21522. doi: 10.1038/s41598-021-00726-4 (PMC8564515; doi:10.1038/s41598-021-00726-4)
Supplement: Supplementary file 1 — Supplementary Information. [file 41598_2021_726_MOESM1_ESM.docx]

**Appendix: Search Strategy**

**PubMed**

(("anti-IL-6"[tw] OR "anti-interleukin-6"[tw] OR "anti-IL6"[tw] OR "anti-interleukin6"[tw] OR "antiIL-6"[tw] OR "antiinterleukin-6"[tw] OR "antiIL6"[tw] OR "antiinterleukin6"[tw] OR "siltuximab"[Supplementary Concept] OR "siltuximab"[tw] OR "siltuximab*"[tw] OR "Sylvant"[tw] OR "CLLB8"[tw] OR "cClB8"[tw] OR "CNTO-328"[tw] OR "CNTO 328"[tw] OR "anti-IL-6R"[tw] OR "anti-interleukin-6R"[tw] OR "anti-IL6R"[tw] OR "anti-interleukin6R"[tw] OR "antiIL-6R"[tw] OR "antiinterleukin-6R"[tw] OR "antiIL6R"[tw] OR "antiinterleukin6R"[tw] OR "tocilizumab"[Supplementary Concept] OR "tocilizumab"[tw] OR "tocilizumab*"[tw] OR "RoActemra"[tw] OR "RHPM-1"[tw] OR "RG-1569"[tw] OR "R-1569"[tw] OR "MSB11456"[tw] OR "MSB-11456"[tw] OR "atlizumab"[tw] OR "MRA monoclonal antibody"[tw] OR "RO-4877533"[tw] OR "Actemra"[tw] OR "sarilumab"[Supplementary Concept] OR "sarilumab"[tw] OR "sarilumab*"[tw] OR "Kevzara"[tw] OR "SAR-153191"[tw] OR "SAR153191"[tw] OR "Kevzara"[tw] OR "REGN-88"[tw] OR "REGN88"[tw])

AND

(("COVID-19"[Supplementary Concept] OR "severe acute respiratory syndrome coronavirus 2"[Supplementary Concept] OR "COVID-19"[all fields] OR "COVID19"[all fields] OR "COVID2019"[all fields] OR "COVID 2019"[all fields] OR "severe acute respiratory syndrome coronavirus 2"[all fields] OR SARS-COV*[all fields] OR SARSCOV*[all fields] OR 2019ncov[all fields] OR "2019 ncov"[all fields] OR novel coronavirus*[all fields] OR novel corona virus*[all fields] OR ((coronavirus*[all fields] OR corona virus*[all fields] OR pneumonia virus*[all fields] OR cov[all fields] OR ncov[all fields]) AND (outbreak[all fields] OR wuhan[all fields] OR "new"[all fields])) OR covid19[all fields] OR "covid 19"[all fields] OR ((coronavirus*[all fields] OR corona virus*[all fields]) AND 2019[all fields]) OR "sars cov 2"[all fields] OR sars2[all fields] OR new coronavirus*[all fields] OR new corona virus*[all fields] OR "ncov 2019"[all fields] OR "sars coronavirus 2"[all fields] OR "sars corona virus 2"[all fields] OR "severe acute respiratory syndrome cov 2"[all fields] OR "severe acute respiratory syndrome cov2"[all fields]) AND ("2019/01/01"[PDAT] : "3000/12/31"[PDAT])))

**PubMed Central (PMC)**

("siltuximab"[Body - Key Terms] OR "siltuximab"[Body - Key Terms] OR "siltuximab*"[Body - Key Terms] OR "Sylvant"[Body - Key Terms] OR "CLLB8"[Body - Key Terms] OR "cClB8"[Body - Key Terms] OR "CNTO-328"[Body - Key Terms] OR "CNTO 328"[Body - Key Terms] OR "tocilizumab"[Body - Key Terms] OR "tocilizumab"[Body - Key Terms] OR "tocilizumab*"[Body - Key Terms] OR "RoActemra"[Body - Key Terms] OR "RHPM-1"[Body - Key Terms] OR "RG-1569"[Body - Key Terms] OR "R-1569"[Body - Key Terms] OR "MSB11456"[Body - Key Terms] OR "MSB-11456"[Body - Key Terms] OR "atlizumab"[Body - Key Terms] OR "MRA monoclonal antibody"[Body - Key Terms] OR "RO-4877533"[Body - Key Terms] OR "Actemra"[Body - Key Terms] OR "sarilumab"[Body - Key Terms] OR "sarilumab"[Body - Key Terms] OR "sarilumab*"[Body - Key Terms] OR "Kevzara"[Body - Key Terms] OR "SAR-153191"[Body - Key Terms] OR "SAR153191"[Body - Key Terms] OR "Kevzara"[Body - Key Terms] OR "REGN-88"[Body - Key Terms] OR "REGN88"[Body - Key Terms])

AND

(("COVID-19"[Supplementary Concept] OR "severe acute respiratory syndrome coronavirus 2"[Supplementary Concept] OR "COVID-19"[Body - Key Terms] OR "COVID19"[Body - Key Terms] OR "COVID2019"[Body - Key Terms] OR "COVID 2019"[Body - Key Terms] OR "severe acute respiratory syndrome coronavirus 2"[Body - Key Terms] OR SARS-COV*[Body - Key Terms] OR SARSCOV*[Body - Key Terms] OR 2019ncov[Body - Key Terms] OR "2019 ncov"[Body - Key Terms] OR novel coronavirus*[Body - Key Terms] OR novel corona virus*[Body - Key Terms] OR ((coronavirus*[Body - Key Terms] OR corona virus*[Body - Key Terms] OR pneumonia virus*[Body - Key Terms] OR cov[Body - Key Terms] OR ncov[Body - Key Terms] ) AND (outbreak[Body - Key Terms] OR wuhan[Body - Key Terms] OR "new"[Body - Key Terms] )) OR covid19[Body - Key Terms] OR "covid 19"[Body - Key Terms] OR ((coronavirus*[Body - Key Terms] OR corona virus*[Body - Key Terms] ) AND 2019[Body - Key Terms] ) OR "sars cov 2"[Body - Key Terms] OR sars2[Body - Key Terms] OR new coronavirus*[Body - Key Terms] OR new corona virus*[Body - Key Terms] OR "ncov 2019"[Body - Key Terms] OR "sars coronavirus 2"[Body - Key Terms] OR "sars corona virus 2"[Body - Key Terms] OR "severe acute respiratory syndrome cov 2"[Body - Key Terms] OR "severe acute respiratory syndrome cov2"[Body - Key Terms] ) AND ("2019/09/01"[PDAT] : "3000/12/31"[PDAT]))

**MEDLINE via OVID**

("anti-IL-6".mp OR "anti-interleukin-6".mp OR "anti-IL6".mp OR "anti-interleukin6".mp OR "antiIL-6".mp OR "antiinterleukin-6".mp OR "antiIL6".mp OR "antiinterleukin6".mp OR "siltuximab"/ OR "siltuximab".mp OR "siltuximab*".mp OR "Sylvant".mp OR "CLLB8".mp OR "cClB8".mp OR "CNTO-328".mp OR "CNTO 328".mp OR "anti-IL-6R".mp OR "anti-interleukin-6R".mp OR "anti-IL6R".mp OR "anti-interleukin6R".mp OR "antiIL-6R".mp OR "antiinterleukin-6R".mp OR "antiIL6R".mp OR "antiinterleukin6R".mp OR "tocilizumab"/ OR "tocilizumab".mp OR "tocilizumab*".mp OR "RoActemra".mp OR "RHPM-1".mp OR "RG-1569".mp OR "R-1569".mp OR "MSB11456".mp OR "MSB-11456".mp OR "atlizumab".mp OR "MRA monoclonal antibody".mp OR "RO-4877533".mp OR "Actemra".mp OR "sarilumab"/ OR "sarilumab".mp OR "sarilumab*".mp OR "Kevzara".mp OR "SAR-153191".mp OR "SAR153191".mp OR "Kevzara".mp OR "REGN-88".mp OR "REGN88".mp)

Limit to COVID-19

**WHO Covid-19 database**

("siltuximab" OR "Sylvant" OR "tocilizumab" OR "RoActemra" OR "atlizumab" OR "Actemra" OR "sarilumab" OR "Kevzara")

("anti-IL-6" OR "anti-interleukin-6" OR "anti-IL6" OR "anti-interleukin6" OR "antiIL-6" OR "antiinterleukin-6" OR "antiIL6" OR "antiinterleukin6" OR "siltuximab" OR "siltuximab" OR "siltuximab*" OR "Sylvant" OR "CLLB8" OR "cClB8" OR "CNTO-328" OR "CNTO 328" OR "anti-IL-6R" OR "anti-interleukin-6R" OR "anti-IL6R" OR "anti-interleukin6R" OR "antiIL-6R" OR "antiinterleukin-6R" OR "antiIL6R" OR "antiinterleukin6R" OR "tocilizumab" OR "tocilizumab" OR "tocilizumab*" OR "RoActemra" OR "RHPM-1" OR "RG-1569" OR "R-1569" OR "MSB11456" OR "MSB-11456" OR "atlizumab" OR "MRA monoclonal antibody" OR "RO-4877533" OR "Actemra" OR "sarilumab" OR "sarilumab" OR "sarilumab*" OR "Kevzara" OR "SAR-153191" OR "SAR153191" OR "Kevzara" OR "REGN-88" OR "REGN88")

**Embase**

("anti-IL-6".mp OR "anti-interleukin-6".mp OR "anti-IL6".mp OR "anti-interleukin6".mp OR "antiIL-6".mp OR "antiinterleukin-6".mp OR "antiIL6".mp OR "antiinterleukin6".mp OR "siltuximab"/ OR "siltuximab".mp OR "siltuximab*".mp OR "Sylvant".mp OR "CLLB8".mp OR "cClB8".mp OR "CNTO-328".mp OR "CNTO 328".mp OR "anti-IL-6R".mp OR "anti-interleukin-6R".mp OR "anti-IL6R".mp OR "anti-interleukin6R".mp OR "antiIL-6R".mp OR "antiinterleukin-6R".mp OR "antiIL6R".mp OR "antiinterleukin6R".mp OR "tocilizumab"/ OR "tocilizumab".mp OR "tocilizumab*".mp OR "RoActemra".mp OR "RHPM-1".mp OR "RG-1569".mp OR "R-1569".mp OR "MSB11456".mp OR "MSB-11456".mp OR "atlizumab".mp OR "MRA monoclonal antibody".mp OR "RO-4877533".mp OR "Actemra".mp OR "sarilumab"/ OR "sarilumab".mp OR "sarilumab*".mp OR "Kevzara".mp OR "SAR-153191".mp OR "SAR153191".mp OR "Kevzara".mp OR "REGN-88".mp OR "REGN88".mp)

AND

(SARS coronavirus/ OR ("COVID-19" OR "severe acute respiratory syndrome coronavirus 2" OR 2019ncov OR "2019 ncov" OR novel coronavirus* OR novel corona virus* OR ((coronavirus* OR corona virus* OR pneumonia virus* OR cov OR ncov) AND (outbreak OR wuhan OR "new")) OR covid19 OR "covid 19" OR ((coronavirus* OR corona virus*) AND 2019) OR "sars cov 2" OR sars2 OR new coronavirus* OR new corona virus* OR "ncov 2019" OR "sars coronavirus 2" OR "sars corona virus 2" OR "severe acute respiratory syndrome cov 2" OR "severe acute respiratory syndrome cov2" OR "COVID-19" OR "COVID19" OR "COVID2019" OR "COVID 2019" OR "severe acute respiratory syndrome coronavirus 2" OR SARS-COV* OR SARSCOV*).af)

AND (2019 OR 2020).yr

**Web of Science**

TS=("anti-IL-6" OR "anti-interleukin-6" OR "anti-IL6" OR "anti-interleukin6" OR "antiIL-6" OR "antiinterleukin-6" OR "antiIL6" OR "antiinterleukin6" OR "siltuximab" OR "siltuximab" OR "siltuximab*" OR "Sylvant" OR "CLLB8" OR "cClB8" OR "CNTO-328" OR "CNTO 328" OR "anti-IL-6R" OR "anti-interleukin-6R" OR "anti-IL6R" OR "anti-interleukin6R" OR "antiIL-6R" OR "antiinterleukin-6R" OR "antiIL6R" OR "antiinterleukin6R" OR "tocilizumab" OR "tocilizumab" OR "tocilizumab*" OR "RoActemra" OR "RHPM-1" OR "RG-1569" OR "R-1569" OR "MSB11456" OR "MSB-11456" OR "atlizumab" OR "MRA monoclonal antibody" OR "RO-4877533" OR "Actemra" OR "sarilumab" OR "sarilumab" OR "sarilumab*" OR "Kevzara" OR "SAR-153191" OR "SAR153191" OR "Kevzara" OR "REGN-88" OR "REGN88")

AND

(TS=("COVID-19" OR "severe acute respiratory syndrome coronavirus 2" OR 2019ncov OR "2019 ncov" OR novel coronavirus* OR novel corona virus* OR ((coronavirus* OR corona virus* OR pneumonia virus* OR cov OR ncov) AND (outbreak OR wuhan OR "new")) OR covid19 OR "covid 19" OR ((coronavirus* OR corona virus*) AND 2019) OR "sars cov 2" OR sars2 OR new coronavirus* OR new corona virus* OR "ncov 2019" OR "sars coronavirus 2" OR "sars corona virus 2" OR "severe acute respiratory syndrome cov 2" OR "severe acute respiratory syndrome cov2" OR "COVID-19" OR "COVID19" OR "COVID2019" OR "COVID 2019" OR "severe acute respiratory syndrome coronavirus 2" OR SARS-COV* OR SARSCOV*) AND py=(2019 OR 2020))

**Cochrane**

**(**("anti-IL-6" OR "anti-interleukin-6" OR "anti-IL6" OR "anti-interleukin6" OR "antiIL-6" OR "antiinterleukin-6" OR "antiIL6" OR "antiinterleukin6" OR "siltuximab" OR "siltuximab" OR "siltuximab*" OR "Sylvant" OR "CLLB8" OR "cClB8" OR "CNTO-328" OR "CNTO 328" OR "anti-IL-6R" OR "anti-interleukin-6R" OR "anti-IL6R" OR "anti-interleukin6R" OR "antiIL-6R" OR "antiinterleukin-6R" OR "antiIL6R" OR "antiinterleukin6R" OR "tocilizumab" OR "tocilizumab" OR "tocilizumab*" OR "RoActemra" OR "RHPM-1" OR "RG-1569" OR "R-1569" OR "MSB11456" OR "MSB-11456" OR "atlizumab" OR "MRA monoclonal antibody" OR "RO-4877533" OR "Actemra" OR "sarilumab" OR "sarilumab" OR "sarilumab*" OR "Kevzara" OR "SAR-153191" OR "SAR153191" OR "Kevzara" OR "REGN-88" OR "REGN88")

AND

("COVID-19" OR "severe acute respiratory syndrome coronavirus 2" OR 2019ncov OR "2019 ncov" OR novel coronavirus* OR novel corona virus* OR ((coronavirus* OR corona virus* OR pneumonia virus* OR cov OR ncov) AND (outbreak OR wuhan OR "new")) OR covid19 OR "covid 19" OR ((coronavirus* OR corona virus*) AND 2019) OR "sars cov 2" OR sars2 OR new coronavirus* OR new corona virus* OR "ncov 2019" OR "sars coronavirus 2" OR "sars corona virus 2" OR "severe acute respiratory syndrome cov 2" OR "severe acute respiratory syndrome cov2" OR "COVID-19" OR "COVID19" OR "COVID2019" OR "COVID 2019" OR "severe acute respiratory syndrome coronavirus 2" OR SARS-COV* OR SARSCOV*)):ti,ab,kw

**Academic Search Premier**

TX("siltuximab" OR "Sylvant" OR "tocilizumab" OR "RoActemra" OR "atlizumab" OR "Actemra" OR "sarilumab" OR "Kevzara")

AND

(TX("COVID-19" OR "severe acute respiratory syndrome coronavirus 2" OR 2019ncov OR "2019 ncov" OR "novel coronavirus*" OR "novel corona virus*") OR TI((coronavirus* OR corona virus* OR pneumonia virus* OR cov OR ncov) AND (outbreak OR wuhan OR "new")) OR TX(covid19 OR "covid 19") OR TI((coronavirus* OR corona virus*) AND 2019) OR TX("sars cov 2" OR sars2 OR "new coronavirus*" OR "new corona virus*" OR "ncov 2019" OR "sars coronavirus 2" OR "sars corona virus 2" OR "severe acute respiratory syndrome cov 2" OR "severe acute respiratory syndrome cov2"))

AND py=(2019 OR 2020)

**Appendix: Risk of bias assessment with MINORS score**

| **Study** | **1*** | **2*** | **3*** | **4*** | **5*** | **6*** | **7*** | **8*** | **9*** | **10*** | **11*** | **12*** | **Total** |
| --- | --- | --- | --- | --- | --- | --- | --- | --- | --- | --- | --- | --- | --- |
| Albertini | 2 | 2 | 1 | 2 | 2 | 2 | 2 | 0 | 2 | 2 | 2 | 2 | 21 |
| Bhadade | 2 | 2 | 1 | 2 | 2 | 0 | 2 | 0 | 2 | 2 | 1 | 2 | 18 |
| Biran | 2 | 2 | 2 | 2 | 2 | 2 | 2 | 0 | 2 | 2 | 2 | 2 | 22 |
| Callejas Rubio | 2 | 1 | 1 | 2 | 2 | 1 | 0 | 0 | 2 | 0 | 0 | 2 | 13 |
| Campochiaro | 2 | 2 | 1 | 2 | 2 | 2 | 2 | 0 | 2 | 2 | 1 | 2 | 20 |
| Canziani | 2 | 2 | 1 | 2 | 2 | 2 | 2 | 0 | 2 | 2 | 2 | 2 | 21 |
| Capra | 2 | 2 | 1 | 2 | 2 | 2 | 2 | 0 | 2 | 2 | 1 | 2 | 20 |
| Carvalho | 2 | 2 | 1 | 2 | 2 | 0 | 2 | 0 | 2 | 2 | 1 | 2 | 18 |
| Chilimuri | 2 | 2 | 1 | 2 | 2 | 2 | 2 | 0 | 2 | 2 | 2 | 2 | 21 |
| Colaneri | 2 | 2 | 1 | 2 | 2 | 1 | 0 | 0 | 2 | 2 | 1 | 2 | 17 |
| De Rossi | 2 | 2 | 1 | 2 | 2 | 2 | 2 | 0 | 2 | 2 | 1 | 2 | 20 |
| Della-Torre | 2 | 2 | 2 | 2 | 2 | 2 | 2 | 0 | 2 | 2 | 1 | 2 | 21 |
| Eimer | 2 | 2 | 1 | 2 | 2 | 2 | 2 | 0 | 2 | 2 | 2 | 2 | 21 |
| Galvan-Roman | 2 | 2 | 1 | 2 | 2 | 2 | 2 | 0 | 2 | 2 | 1 | 2 | 20 |
| Garcia | 2 | 2 | 2 | 2 | 2 | 2 | 2 | 0 | 2 | 2 | 0 | 2 | 20 |
| Gokhale | 2 | 2 | 1 | 2 | 2 | 2 | 2 | 0 | 2 | 2 | 1 | 2 | 20 |
| Gritti | 2 | 2 | 2 | 2 | 2 | 2 | 2 | 0 | 2 | 2 | 2 | 2 | 22 |
| Guaraldi | 2 | 2 | 1 | 2 | 2 | 2 | 2 | 0 | 2 | 2 | 1 | 2 | 20 |
| Guisado-Vasco | 2 | 2 | 1 | 2 | 2 | 2 | 2 | 0 | 2 | 2 | 0 | 2 | 19 |
| Gupta | 2 | 2 | 2 | 2 | 2 | 2 | 2 | 0 | 2 | 2 | 2 | 2 | 22 |
| Hill | 2 | 2 | 1 | 2 | 2 | 2 | 2 | 0 | 2 | 2 | 1 | 2 | 20 |
| Holt | 2 | 1 | 1 | 2 | 2 | 2 | 2 | 0 | 2 | 2 | 0 | 2 | 18 |
| IP | 2 | 2 | 1 | 2 | 2 | 2 | 2 | 0 | 2 | 2 | 2 | 2 | 21 |
| Kewan | 2 | 2 | 1 | 2 | 2 | 2 | 2 | 0 | 2 | 2 | 1 | 2 | 20 |
| Kimmig | 2 | 2 | 1 | 2 | 2 | 0 | 2 | 0 | 2 | 2 | 1 | 2 | 18 |
| King | 2 | 2 | 1 | 2 | 2 | 0 | 2 | 0 | 2 | 2 | 1 | 2 | 18 |
| Klopfenstein | 2 | 2 | 1 | 2 | 2 | 0 | 2 | 0 | 2 | 2 | 1 | 2 | 18 |
| Klopfenstein | 2 | 2 | 1 | 2 | 2 | 0 | 2 | 0 | 2 | 2 | 1 | 2 | 18 |
| Lewis | 2 | 2 | 1 | 2 | 2 | 2 | 2 | 0 | 2 | 2 | 1 | 2 | 20 |
| Li | 2 | 2 | 1 | 2 | 2 | 0 | 2 | 0 | 2 | 2 | 1 | 2 | 18 |
| López-Medrano | 2 | 2 | 1 | 2 | 2 | 2 | 2 | 0 | 2 | 2 | 2 | 2 | 21 |
| Martin-Moro | 1 | 1 | 1 | 2 | 2 | 2 | 2 | 0 | 2 | 2 | 1 | 2 | 18 |
| Martínez-Sanz | 2 | 2 | 1 | 2 | 2 | 2 | 2 | 0 | 2 | 2 | 1 | 2 | 20 |
| Masia | 2 | 2 | 1 | 2 | 2 | 2 | 2 | 0 | 2 | 2 | 1 | 2 | 20 |
| Menzella | 2 | 2 | 1 | 2 | 2 | 2 | 2 | 0 | 2 | 2 | 1 | 2 | 20 |
| Mikulska | 2 | 2 | 1 | 2 | 2 | 2 | 2 | 0 | 2 | 2 | 1 | 2 | 20 |
| Moiseev | 2 | 2 | 1 | 2 | 2 | 0 | 2 | 0 | 2 | 2 | 1 | 2 | 18 |
| Moreno-García | 2 | 1 | 1 | 2 | 2 | 2 | 2 | 0 | 2 | 2 | 1 | 2 | 19 |
| Narain | 2 | 1 | 1 | 2 | 2 | 2 | 2 | 0 | 2 | 2 | 1 | 2 | 19 |
| Okoh | 2 | 1 | 1 | 2 | 2 | 2 | 2 | 0 | 2 | 2 | 1 | 2 | 19 |
| Pereira | 2 | 1 | 1 | 2 | 2 | 2 | 2 | 0 | 2 | 2 | 1 | 2 | 19 |
| Petrak | 2 | 2 | 1 | 2 | 2 | 0 | 2 | 0 | 2 | 2 | 2 | 2 | 19 |
| Pettit | 2 | 2 | 1 | 2 | 2 | 2 | 2 | 0 | 2 | 2 | 1 | 2 | 20 |
| Piano | 1 | 1 | 1 | 2 | 2 | 0 | 2 | 0 | 2 | 2 | 1 | 2 | 16 |
| Potere | 2 | 2 | 1 | 2 | 2 | 2 | 2 | 0 | 2 | 2 | 1 | 2 | 20 |
| Ramaswamy | 2 | 2 | 1 | 2 | 2 | 1 | 2 | 0 | 2 | 2 | 1 | 2 | 19 |
| Rivera-Izquierdo | 2 | 2 | 1 | 2 | 2 | 2 | 2 | 0 | 2 | 2 | 0 | 2 | 19 |
| Rodriguez-Bano | 2 | 2 | 1 | 2 | 2 | 2 | 2 | 0 | 2 | 2 | 1 | 2 | 20 |
| Rojas-Marte | 2 | 2 | 1 | 2 | 2 | 2 | 2 | 0 | 2 | 2 | 1 | 2 | 20 |
| Roomi | 2 | 2 | 1 | 2 | 2 | 1 | 2 | 0 | 2 | 2 | 1 | 2 | 19 |
| Rossi | 2 | 2 | 1 | 2 | 2 | 2 | 2 | 0 | 2 | 2 | 2 | 2 | 21 |
| Rossotti | 2 | 2 | 1 | 2 | 2 | 2 | 2 | 0 | 2 | 2 | 1 | 2 | 20 |
| Roumier | 2 | 2 | 1 | 2 | 2 | 2 | 2 | 1 | 2 | 2 | 1 | 2 | 21 |
| Roumier | 2 | 2 | 1 | 2 | 2 | 1 | 2 | 0 | 2 | 2 | 1 | 2 | 19 |
| Ruiz-Antoran | 2 | 2 | 1 | 2 | 2 | 2 | 2 | 0 | 2 | 2 | 2 | 2 | 21 |
| Salvati | 2 | 2 | 1 | 2 | 2 | 0 | 2 | 0 | 2 | 2 | 1 | 2 | 18 |
| Somers | 2 | 2 | 2 | 2 | 2 | 2 | 2 | 0 | 2 | 2 | 1 | 2 | 21 |
| Taboada | 2 | 2 | 2 | 2 | 2 | 2 | 2 | 0 | 2 | 2 | 0 | 2 | 20 |
| Tsai | 2 | 1 | 1 | 2 | 2 | 0 | 2 | 0 | 2 | 2 | 2 | 2 | 18 |
| Vena | 2 | 2 | 2 | 2 | 2 | 0 | 2 | 1 | 2 | 2 | 0 | 2 | 19 |
| Wadud | 2 | 2 | 1 | 2 | 2 | 0 | 2 | 0 | 2 | 2 | 0 | 1 | 16 |
| Zantah | 2 | 2 | 1 | 2 | 2 | 2 | 2 | 0 | 2 | 2 | 1 | 2 | 20 |

*1. A stated aim of the study;

2. inclusion of consecutive patients;

3. prospective collection of data;

4. endpoints appropriate to study aim;

5. unbiased assessment of study endpoint;

6. Follow-up period appropriate to the major endpoint;

7. Less than 5% lost to follow-up;

8.; adequate control group

9. contemporary groups;

10. baseline equivalence of groups;

11. prospective calculation of study size;

12. adequate statistical analyses

**Appendix: Risk of bias assessment with RoB2 score.**

| **Study** | **1*** | **2*** | **3*** | **4*** | **5*** |
| --- | --- | --- | --- | --- | --- |
| Salvarani | 0 | 0 | 1 | 1 | 1 |
| Stone | 0 | 1 | 1 | 1 | 1 |
| Zhao | 0 | 0 | 1 | 1 | 1 |
| Gordon (TCZ) | 1 | 0 | 1 | 1 | 1 |
| Gordon (SAR) | 1 | 0 | 1 | 1 | 1 |
| Hermine | 1 | 0 | 1 | 1 | 1 |
| Horby | 1 | 0 | 1 | 1 | 1 |
| Rosas | 1 | 0 | 1 | 1 | 1 |
| Salama | 1 | 0 | 1 | 1 | 1 |
| Veiga | 1 | 0 | 1 | 1 | 1 |

*1 bias arising from the randomization process

2 bias due to deviations from the intended interventions

3 bias due to missing outcome data

4 bias in measurement of the outcome

5 bias in selection of the reported results

0 = some concerns 1= low risk of bias

TCZ = tocilizumab SAR = sarilumab

**Appendix: forest plots**

Figure S1: Forest plot for mortality

Figure S2: Forest plot mechanical ventilation

Figure S3: Forest plot for ICU admission

Figure S4: Forest plot for composite of mortality and mechanical ventilation

Figure S5: Forest plot for secondary infection

Figure S6: Forest plot for neutropenia

Figure S7: Forest plot for impaired liver function

Figure S8: Forest plot for pulmonary embolism

Metafor R package was used to generate this figures[13, 23].

13. Viechtbauer W. Conducting meta-analyses in R with the metafor package. Journal of Statistical Software 2010; 36(3): 1-48.

23. The R Core team. R: A language and Environment for Statistical Computing, R foundation for

Statistical Computing (Vienna Austria), 2021, https://www.R-project.org.

**Figure S1**

**
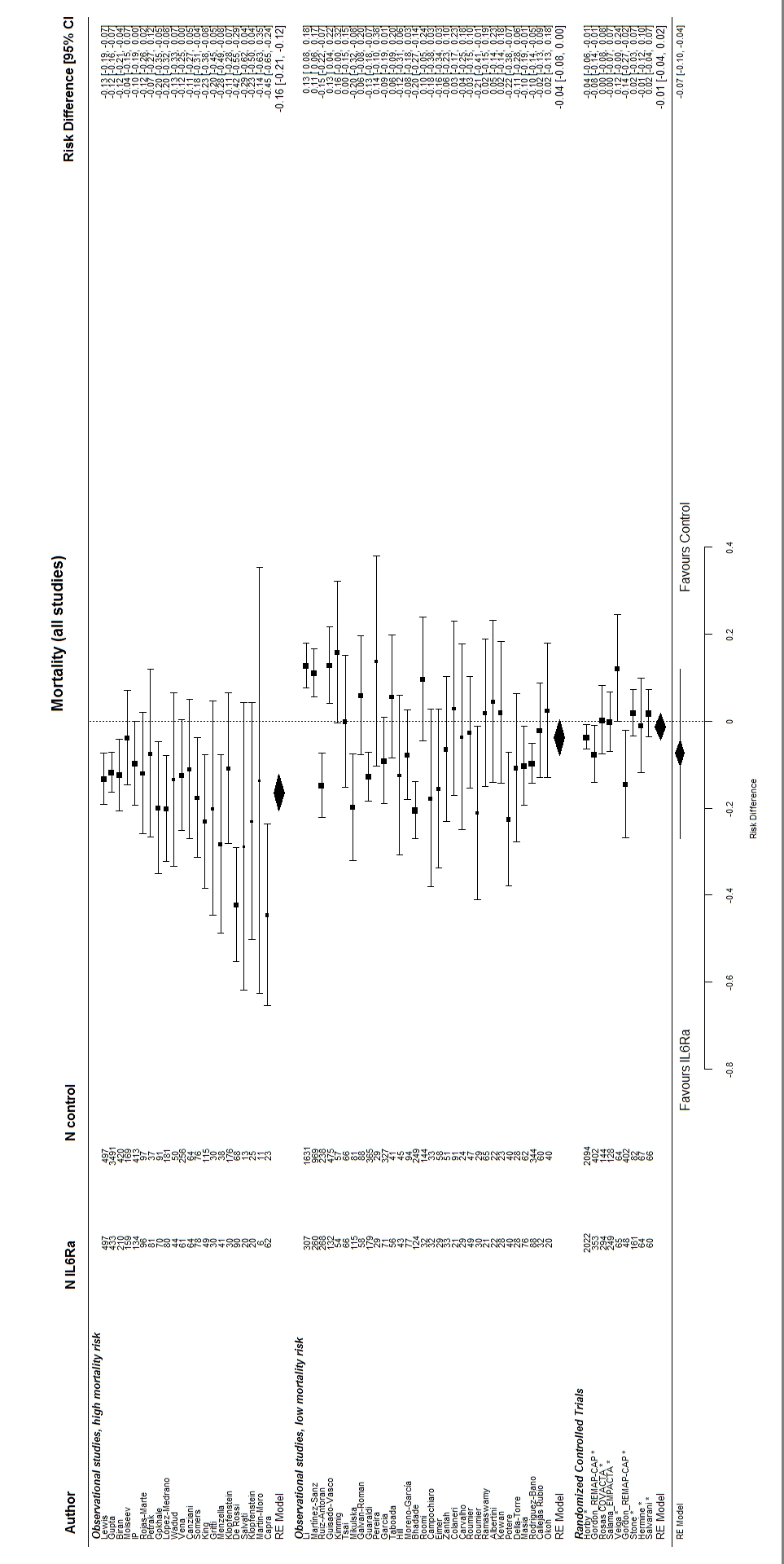
**

Figure S1: Forest plot showing the risk difference in mortality between patients treated with IL-6 (receptor) antagonists and patients not treated with IL-6 (receptor) antagonists. I2 was 82%. Metafor R package was used to generate this figure[13,23].

IL6Ra = IL-6 (receptero) antagonist

TCZ = tocilizumab

SAR = sarilumab

* = RCT

**Figure S2**

**
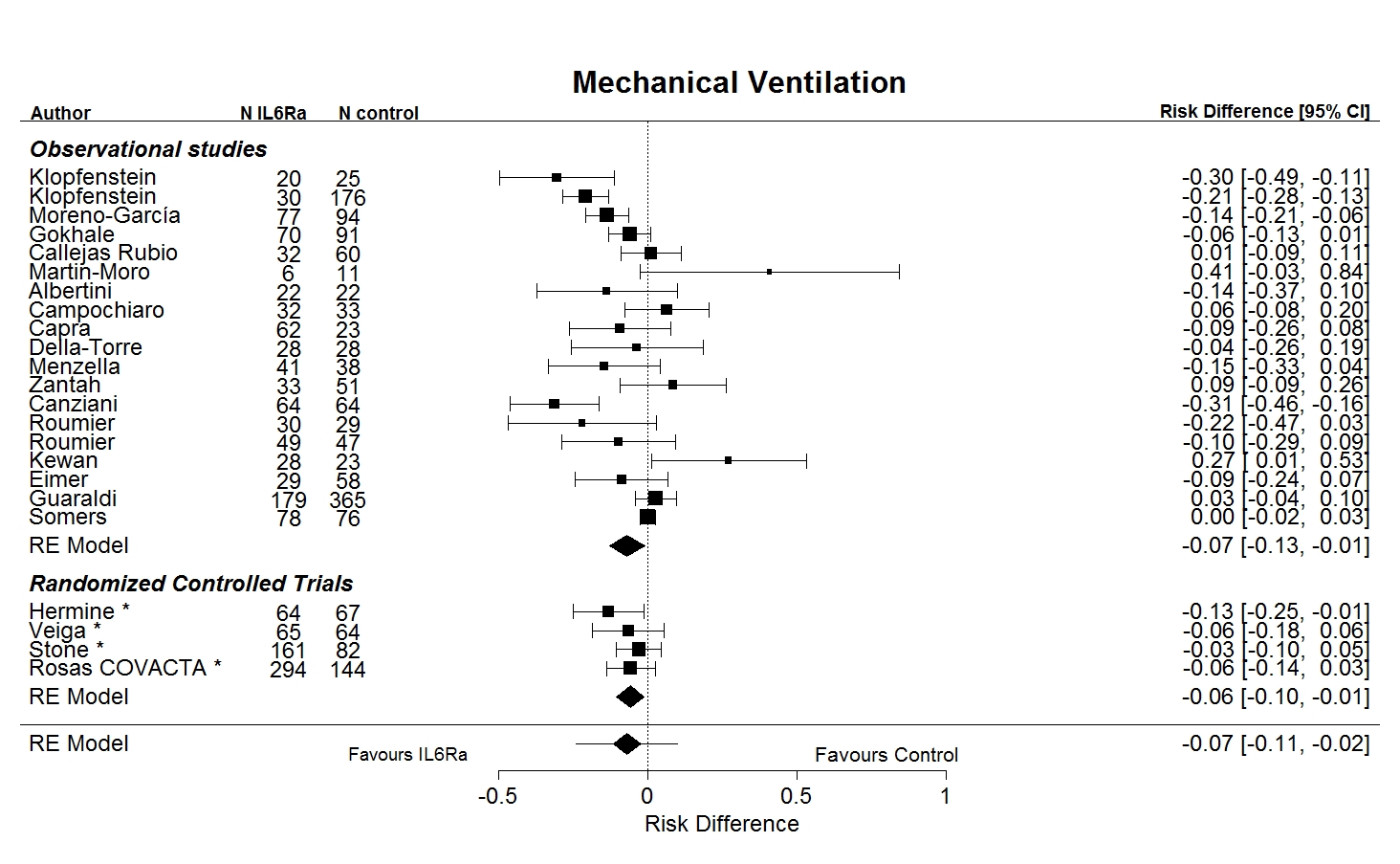
**

Figure S2: Forest plot showing the risk difference in mechanical ventilation between patients treated with IL-6 (receptor) antagonists and patients not treated with IL-6 (receptor) antagonists. I2 was 71%. Metafor R package was used to generate this figure[13,23].

IL6Ra = IL-6 (receptero) antagonist

TCZ = tocilizumab

SAR = sarilumab

* = RCT

**Figure S3**

**
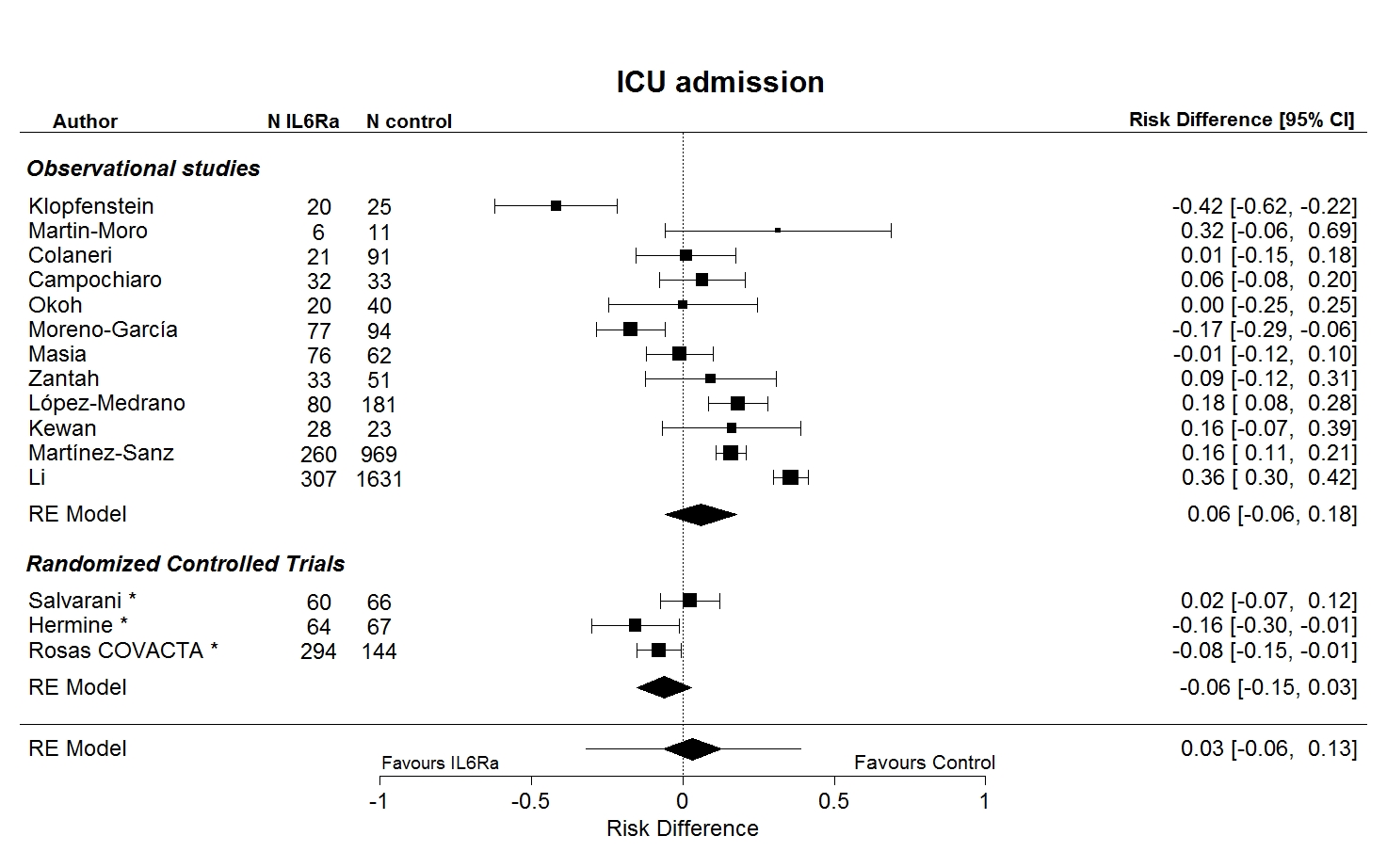
**

Figure S3: Forest plot showing the risk difference in ICU admission between patients treated with IL-6 (receptor) antagonists and patients not treated with IL-6 (receptor) antagonists. I2 was 91%. Metafor R package was used to generate this figure[13,23].

IL6Ra = IL-6 (receptero) antagonist

TCZ = tocilizumab

SAR = sarilumab

* = RCT

**Figure S4**

**
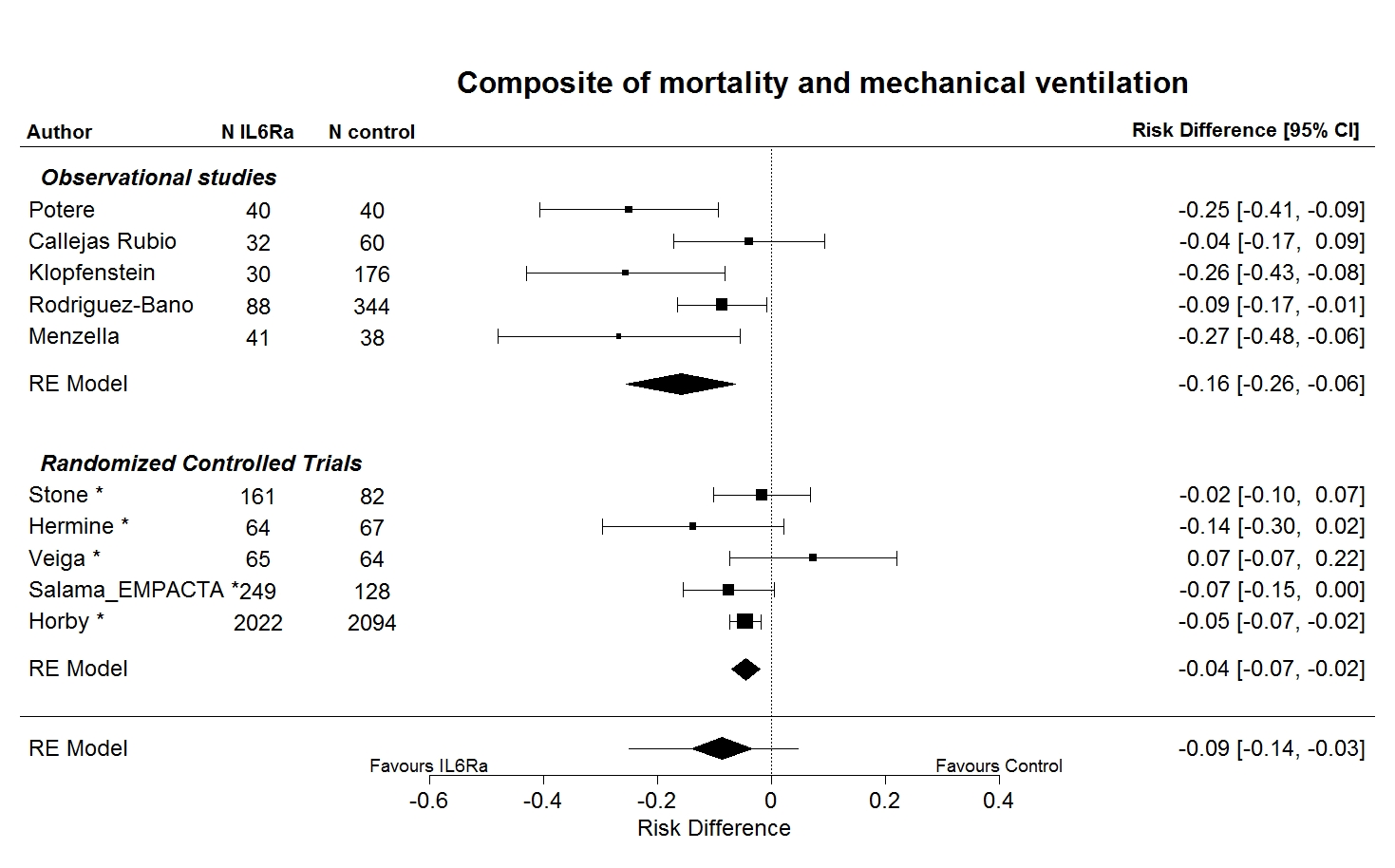
**

Figure S4: Forest plot showing the risk difference in composite of mortality and mechanical ventilation between patients treated with IL-6 (receptor) antagonists and patients not treated with IL-6 (receptor) antagonists. I2 was 47%. Metafor R package was used to generate this figure[13,23].

IL6Ra = IL-6 (receptero) antagonist

TCZ = tocilizumab

SAR = sarilumab

* = RCT

**Figure S5**

**
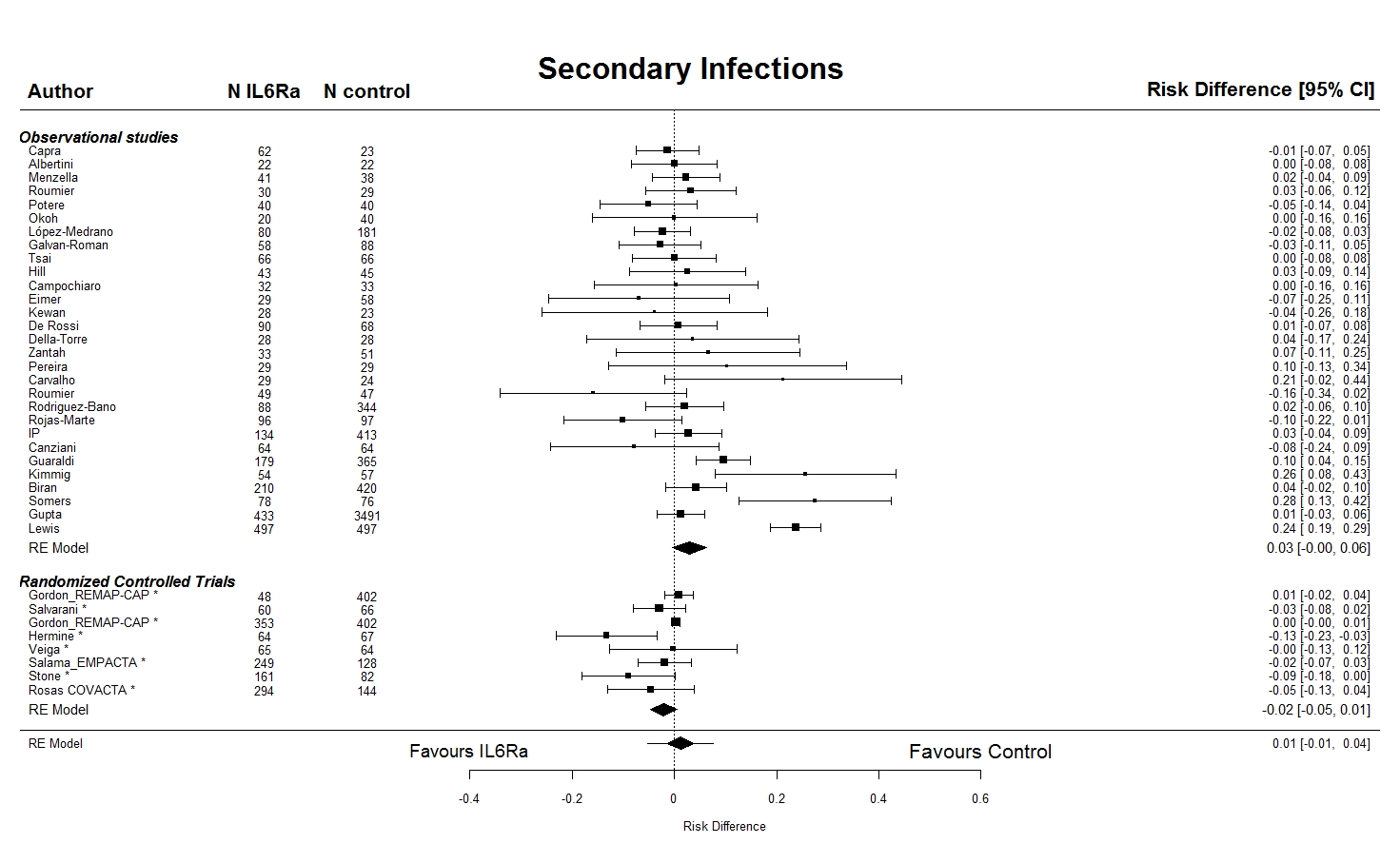
**

Figure S5: Forest plot showing the risk difference in secondary infections between patients treated with IL-6 (receptor) antagonists and patients not treated with IL-6 (receptor) antagonists. I2 was 86%. Metafor R package was used to generate this figure[13,23].

IL6Ra = IL-6 (receptero) antagonist

TCZ = tocilizumab

SAR = sarilumab

* = RCT

**Figure S6**

**
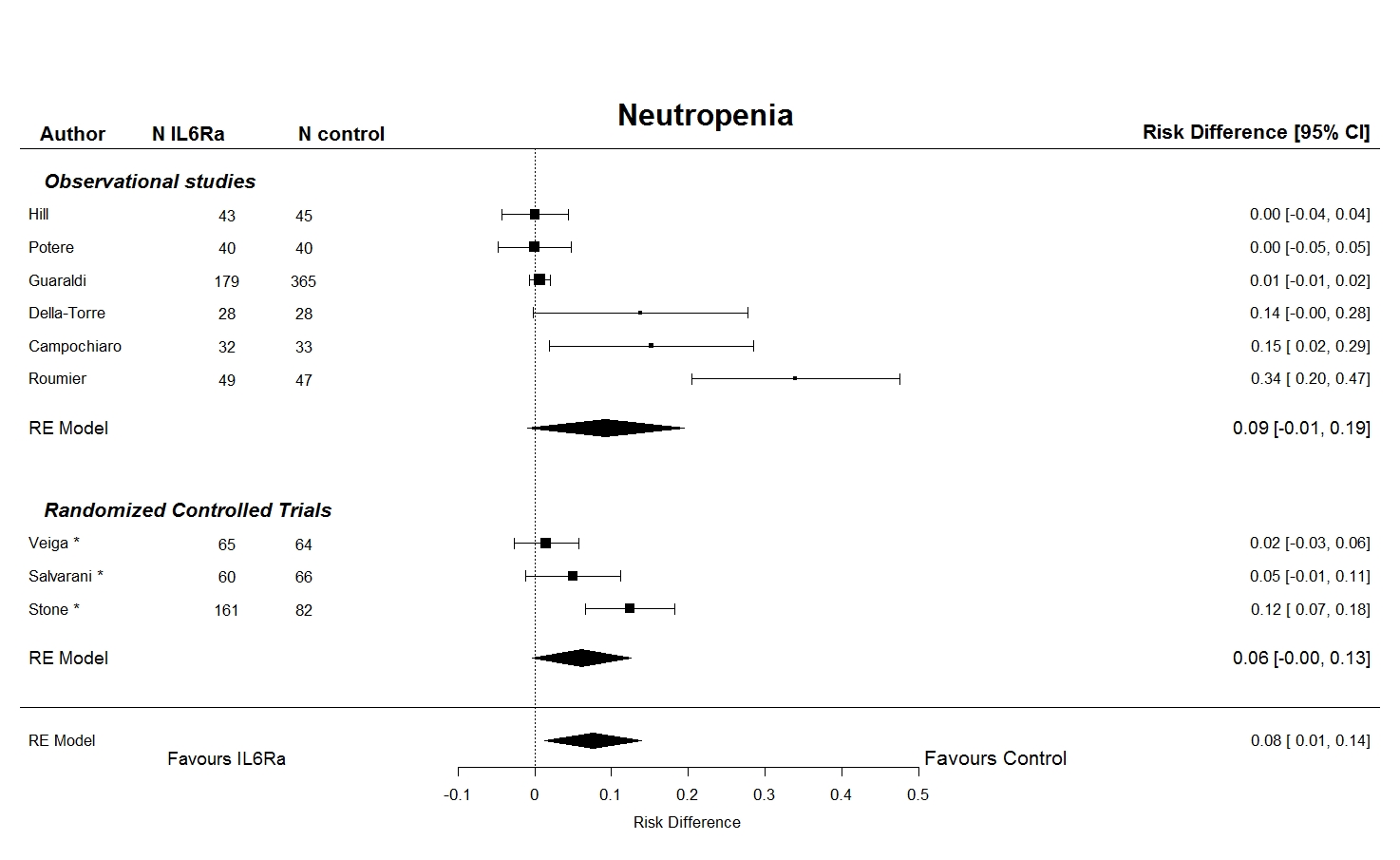
**

Figure S6: Forest plot showing the risk difference in secondary infections between patients treated with IL-6 (receptor) antagonists and patients not treated with IL-6 (receptor) antagonists. I2 was 0%. Metafor R package was used to generate this figure[13,23].

IL6Ra = IL-6 (receptero) antagonist

TCZ = tocilizumab

SAR = sarilumab

* = RCT

**Figure S7**

**
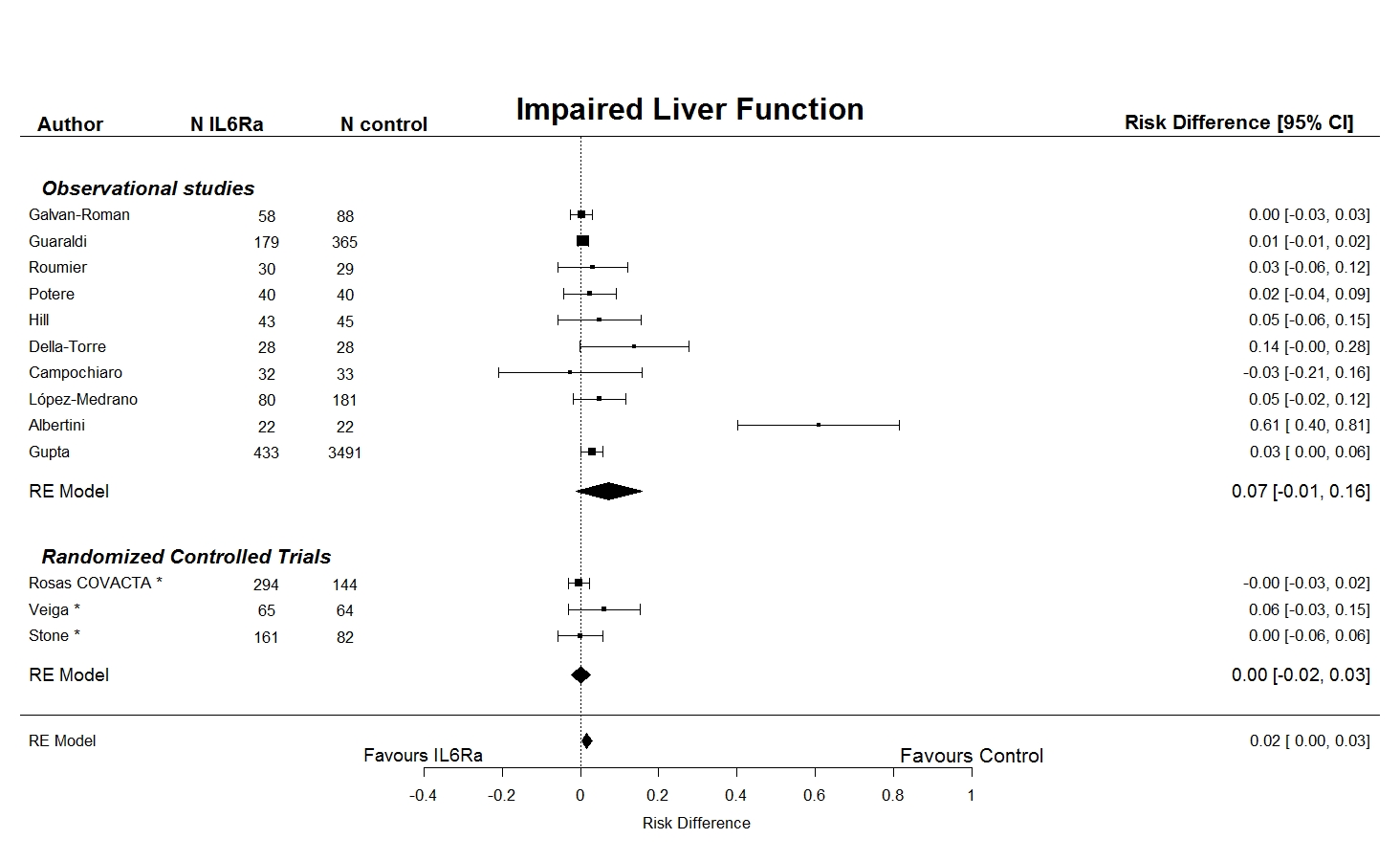
**

Figure S7: Forest plot showing the risk difference in impaired liver function between patients treated with IL-6 (receptor) antagonists and patients not treated with IL-6 (receptor) antagonists. I2 was 0%. Metafor R package was used to generate this figure[13,23].

IL6Ra = IL-6 (receptero) antagonist

TCZ = tocilizumab

SAR = sarilumab

* = RCT

**Figure S8**

**
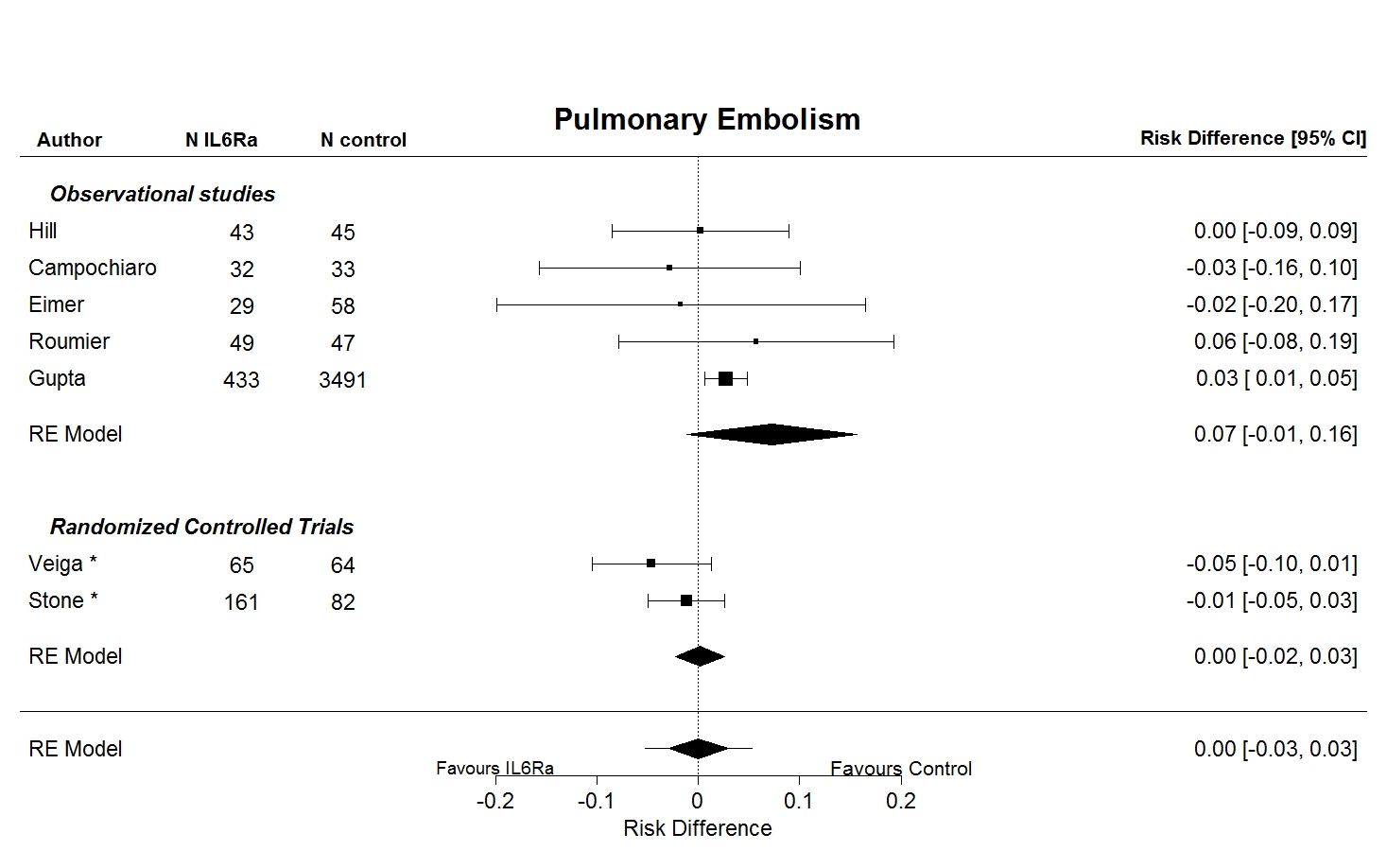
**

Figure S8: Forest plot showing the risk difference in pulmonary embolism between patients treated with IL-6 (receptor) antagonists and patients not treated with IL-6 (receptor) antagonists. I2 was 38%. Metafor R package was used to generate this figure[13,23].

IL6Ra = IL-6 (receptero) antagonist

TCZ = tocilizumab

SAR = sarilumab

* = RCT

**Appendix: references of included studies**

1. Albertini L, Soletchnik M, Razurel A, et al. Observational study on off-label use of tocilizumab in patients with severe COVID-19. Eur J Hosp Pharm, 2021 ; 28: 22-27.

2. Bhadade R, Harde M, deSouza R, et al. Appraisal of critically ill COVID-19 patients at a dedicated COVID hospital. J Assoc Physicians India, 2020 ; 68:14-19.

3. Biran N, Ip A, Ahn J, et al. Tocilizumab among patients with COVID-19 in the intensive care unit: a multicentre observational study. The Lancet Rheumatology, 2020 ; 2: e603-e612.

4. Callejas Rubio JL, Luna Del Castillo JD, de la Hera Fernández J, Guirao Arrabal E, Colmenero Ruiz M, Ortego Centeno N. Effectiveness of corticoid pulses in patients with cytokine storm syndrome induced by SARS-CoV-2 infection. Med Clin (Barc), 2020 ;155: 159-161.

5. Campochiaro C, Della-Torre E, Cavalli G, et al. Efficacy and safety of tocilizumab in severe COVID-19 patients: a single-centre retrospective cohort study. Eur J Intern Med, 2020 ; 76: 43- 9.

6. Canziani LM, Trovati S, Brunetta E, et al. Interleukin-6 receptor blocking with intravenous tocilizumab in COVID-19 severe acute respiratory distress syndrome: A retrospective case-control survival analysis of 128 patients. J Autoimmun, 2020 ; 114: 102511.

7. Capra R, De Rossi N, Mattioli F, et al. Impact of low dose tocilizumab on mortality rate in patients with COVID-19 related pneumonia. Eur J Intern Med, 2020 ; 76: 31-5.

8. Carvalho V, Turon R, Goncalves B, et al. Effects of tocilizumab in critically ill patients with COVID-19: A quasi-experimental study. MedRxiv 2020.07.13.20149328 [preprint]. July 15, 2020 [cited 2021 Jan 20]. Available from: https://doi.org/10.1101/2020.07.13.20149328.

9. Chilimuri S, Sun H, Alemam A, et al. Tocilizumab use in patients with moderate to severe COVID‐19: A retrospective cohort study. J Clin Pharm Ther, 2020 ; 00: 1– 7

10. Colaneri M, Bogliolo L, Valsecchi P, et al. Tocilizumab for treatment of severe COVID-19 patients: Preliminary results from SMAtteo COvid19 REgistry (SMACORE). Microorganisms, 2020 ; 8: 695.

11. De Rossi N, Scarpazza C, Filippini C, et al. Early use of low dose tocilizumab in patients with COVID-19: A retrospective cohort study with a complete follow-up. EClinicalMedicine, 2020 ; 25: 100459.

12. Della-Torre E, Campochiaro C, Cavalli G, et al. Interleukin-6 blockade with sarilumab in severe COVID-19 pneumonia with systemic hyperinflammation: an open-label cohort study. Ann Rheum Dis, 2020 ; 79: 1277-1285.

13. Eimer J, Vesterbacka J, Svensson AK, et al. Tocilizumab shortens time on mechanical ventilation and length of hospital stay in patients with severe COVID-19: a retrospective cohort study. J Intern Med, 2021 ; 289: 434-436.

14.Wendel Garcia PD, Fumeaux T, Guerci P, et al. Prognostic factors associated with mortality risk and disease progression in 639 critically ill patients with COVID-19 in Europe: Initial report of the international RISC-19-ICU prospective observational cohort. EClinicalMedicine, 2020 ; 25: 100449.

15. Gokhale Y, Mehta R, Karnik N, Kulkarni U, Gokhale S. Tocilizumab improves survival in patients with persistent hypoxia in severe COVID-19 pneumonia. EClinicalMedicine, 2020 ; 24: 100467.

16. Gordon AC, Mouncey PR, Al-Beidh F, et al. Interleukin-6 Receptor antagonists in critically ill patients with COVID-19 – Preliminary report. MedRxiv 2021.2001.2007.21249390 [Preprint]. Jan 7, 2021 [cited 2021 Jan 20]. Available from: https://doi.org/10.1101/2021.01.07.21249390.

17. Gritti G, Raimondi F, Ripamonti D, et al. IL-6 signalling pathway inactivation with siltuximab in patients with COVID-19 respiratory failure: an observational cohort study. MedRxiv 2020.2004.2001.20048561 [Preprint]. June 20, 2020 [cited 2021 Jan 20]. Available from: https://doi.org/10.1101/2020.04.01.20048561.

18. Guaraldi G, Meschiari M, Cozzi-Lepri A, et al. Tocilizumab in patients with severe COVID-19: a retrospective cohort study. The Lancet Rheumatology, 2020 ; 2: e474-e484.

19. Guisado-Vasco P, Valderas-Ortega S, Maravillas Carralón-González M, et al. Clinical characteristics and outcomes among hospitalized adults with severe COVID-19 admitted to a tertiary medical center and receiving antiviral, antimalarials, glucocorticoids, or immunomodulation with tocilizumab or cyclosporine: A retrospective observational study (COQUIMA cohort).

EClinicalMedicine, 2020 ; 28: 100591.

20. Gupta S, Wang W, Hayek SS, et al. Association between early treatment with tocilizumab and mortality among critically ill patients with COVID-19. JAMA Intern Med, 2021 ; 181: 41-51.

21. Hermine O, Mariette X, Tharaux PL, Resche-Rigon M, Porcher R, Ravaud P. Effect of tocilizumab vs usual care in adults hospitalized with COVID-19 and moderate or severe pneumonia: A randomized clinical trial. JAMA Intern Med, 2021 ; 181: 32-40.

22. Hill JA, Menon MP, Dhanireddy S, et al. Tocilizumab in hospitalized patients with COVID-19: Clinical outcomes, inflammatory marker kinetics, and safety. J Med Virol, 2021 ; 93: 2270-2280.

23. Holt GE, Batra M, Murthi M, et al. Lack of tocilizumab effect on mortality in COVID19 patients. Sci Rep, 2020 ; 10: 17100.

24. Horby PW, Pessoa-Amorim G, Peto L, et al. Tocilizumab in patients admitted to hospital with COVID-19 (RECOVERY): preliminary results of a randomised, controlled, open-label, platform trial. MedRxiv 2021.2002.2011.21249258 [Preprint]. Feb 11, 2021 [cited 2021 Jan 20]. Available from: https://doi.org/10.1101/2021.02.11.21249258

25. Ip A, Berry DA, Hansen E, et al. Hydroxychloroquine and tocilizumab therapy in COVID-19 patients—An observational study. PLOS ONE, 2020 ; 15: e0237693.

26. Kewan T, Covut F, Al–Jaghbeer MJ, Rose L, Gopalakrishna K, Akbik B. Tocilizumab for treatment of patients with severe COVID–19: A retrospective cohort study. EClinicalMedicine, 2020 ; 24: 100418.

27. Kimmig LM, Wu D, Gold M, et al. IL-6 inhibition in critically ill COVID-19 patients is associated with increased secondary infections. Frontiers in Medicine, 2020 ; 7: 583897.

28. King CS, Sahjwani D, Whitney Brown A, et al. Outcomes of mechanically ventilated patients with COVID-19 associated respiratory failure. PLoS One, 2020 ; 15: 1-9.

29. Klopfenstein T, Zayet S, Lohse, A, et al. Impact of tocilizumab on mortality and/or invasive mechanical ventilation requirement in a cohort of 206 COVID-19 patients. Int J Infect Dis, 2020 ; 99: 491-495.

30. Klopfenstein T, Zayet S, Lohse A, et al. Tocilizumab therapy reduced intensive care unit admissions and/or mortality in COVID-19 patients. Med Mal Infect, 2020 ; 50: 397-400.

31. Lewis TC, Adhikari S, Tatapudi V, et al. A propensity-matched cohort study of tocilizumab in patients with coronavirus disease 2019. Critical Care Explorations, 2020 ; 2: e0283.

32. Li M, Yoo EJ, Baram M, et al. Tocilizumab in the management of COVID-19: A preliminary report. Am J Med Sci, 2021 ; 36: 208-215.

33. López-Medrano F, Pérez-Jacoiste Asín MA, Fernández-Ruiz M, et al. Combination therapy with tocilizumab and corticosteroids for aged patients with severe COVID-19 pneumonia: a single-center retrospective study. MedRxiv 2020.09.26.20202283 [Preprint]. Sep 30, 2020 [cited 2021 Jan 20]. Available from: https://doi.org/10.1101/2020.09.26.20202283.

34. Martín-Moro F, Marquet J, Piris M, et al. Survival study of hospitalised patients with concurrent COVID-19 and haematological malignancies. Br J Haematol, 2020 ; 190: e16- e20.

35. Martínez-Sanz J, Muriel A, Ron R, et al. Effects of tocilizumab on mortality in hospitalized patients with COVID-19: a multicentre cohort study. Clin Microbiol Infect, 2021 ; 27: 238-243.

36. Masiá M, Fernández-González M, Padilla S, et al. Impact of interleukin-6 blockade with tocilizumab on SARS-CoV-2 viral kinetics and antibody responses in patients with COVID-19: A prospective cohort study. EBioMedicine, 2020 ; 60: 102999.

37. Menzella F, Fontana M, Salvarani C, et al. Efficacy of tocilizumab in patients with COVID-19 ARDS undergoing noninvasive ventilation. Crit Care, 2020 ; 24: 589.

38. Mikulska M, Nicolini LA, Signori A, et al. Tocilizumab and steroid treatment in patients with COVID-19 pneumonia. PLoS One, 2020 ; 15: e0237831.

39. Moiseev S, Avdeev S, Tao E, et al. Neither earlier nor late tocilizumab improved outcomes in the intensive care unit patients with COVID-19 in a retrospective cohort study. Ann Rheum Dis, 2020 [Online ahead of print]. Available from: https://doi.org/10.1136/annrheumdis-2020-219265.

40. Moreno-García E, Rico V, Albiach L, et al. Tocilizumab is associated with reduced risk of ICU admission and mortality in patients with SARS-CoV-2 infection. MedRxiv 2020.2006.2005.20113738 [Preprint]. June 5 2020 [cited 2021 Jan 20]. Available from: https://doi.org/10.1101/2020.06.05.20113738.

41. Narain S, Stefanov DG, Chau AS, et al. Comparative Survival Analysis of Immunomodulatory Therapy for Coronavirus Disease 2019 Cytokine Storm. Chest, 2020 [Online ahead of print]. Available from: https://doi.org/10.1016/j.chest.2020.09.275.

42. Okoh AK, Bishburg E, Grinberg S, Nagarakanti S. Tocilizumab use in COVID‐19‐associated pneumonia. J Med Virol, 2021 ; 93: 1023‐1028.

43. Pereira MR, Aversa MM, Farr MA, et al. Tocilizumab for severe COVID-19 in solid organ transplant recipients: a matched cohort study. Am J Transplant, 2020 ; 20: 3198-3205.

44. Petrak RM, Van Hise NW, Skorodin NC, et al. Early tocilizumab dosing is associated with improved survival in critically ill patients infected with Sars-CoV-2. MedRxiv 2020.2010.2027.20211433 [Preprint]. Oct 28, 2020 [cited 2021 Jan 20]. Available from: https://doi.org/10.1101/2020.10.27.20211433.

45. Pettit NN, Nguyen CT, Mutlu GM, et al. Late onset infectious complications and safety of tocilizumab in the management of COVID-19. J Med Virol, 2021 ; 93: 1459-1464.

46. Piano S, Vettor R, Angeli P. Tocilizumab for severe COVID-19 pneumonia. Lancet Rheumatol, 2020 ; 2: e659-e660.

47. Potere N, Di Nisio M, Cibelli D, et al. Interleukin-6 receptor blockade with subcutaneous tocilizumab in severe COVID-19 pneumonia and hyperinflammation: a case– control study. Ann Rheum Dis, 2021 ; 80: 271–272.

48. Ramaswamy M, Mannam P, Comer R, Sinclair E, McQuaid DB, Schmidt ML. Off-Label Real World Experience Using Tocilizumab for Patients Hospitalized with COVID-19 Disease in a Regional Community Health System: A Case-Control Study. MedRxiv 2020.2005.2014.20099234 [Preprint]. May 19 2020 [cited 2021 Jan 20]. Available from: https://doi.org/10.1101/2020.05.14.20099234.

49. Rivera-Izquierdo M, el Carmen Valero-Ubierna M, R-delAmo JL,

et al. Therapeutic agents tested in 238 COVID-19 hospitalized patients and their relationship with mortality. Med Clin (Barc), 2020 ; 155: 375-381.

50. Rodríguez-Baño J, Pachón J, Carratalà J, et al. Treatment with tocilizumab or corticosteroids for COVID-19 patients with hyperinflammatory state: a multicentre cohort study (SAM-COVID-19). Clin Microbiol Infect, 2021 ; 27: 244-252.

51. Rojas-Marte GR, Khalid M, Mukhtar O, et al. Outcomes in Patients with Severe COVID-19 Disease Treated with Tocilizumab - A Case- Controlled Study. Qjm, 2020 ; 113: 546-550.

52. Roomi S, Ullah W, Ahmed F, et al. Efficacy of Hydroxychloroquine and Tocilizumab in Patients With COVID-19: Single-Center Retrospective Chart Review. J Med Internet Res, 2020 ; 22: e21758.

53. Rosas IO, Bräu N, Waters M, et al. Tocilizumab in Hospitalized Patients with Severe Covid-19 Pneumonia. N Engl J Med, 2021.

54. Rossi B, Nguyen LS, Zimmermann P, et al. Effect of tocilizumab in hospitalized patients with severe COVID-19 pneumonia: A case-control cohort study. Pharmaceuticals, 2020 ; 13: 317.

55. Rossotti R, Travi G, Ughi N, et al. Safety and efficacy of anti-il6-receptor tocilizumab use in severe and critical patients affected by coronavirus disease 2019: A comparative analysis. J Infect, 2020 ; 81: e11-e17.

56. Roumier, Paule R, Groh M, et al. Interleukin-6 blockade for severe COVID-19. MedRxiv 2020.2004.2020.20061861 [Preprint]. Apr 22, 2020 [cited 2021 Jan 20]. Available from: https://doi.org/10.1101/2020.04.20.20061861.

57. Roumier M, Paule R, Vallée A, et al. Tocilizumab for severe worsening COVID-19 pneumonia: a propensity score analysis. J Clin Immunol, 2021 ; 41: 303-314.

58. Ruiz-Antorán B, Sancho-López A, Torres F, et al. Combination of Tocilizumab and Steroids to Improve Mortality in Patients with Severe COVID-19 Infection: A Spanish, Multicenter, Cohort Study. Infectious Diseases and Therapy, 2020 ; 6: 1–16.

59. Salama C, Han J, Yau L, et al. Tocilizumab in Patients Hospitalized with Covid-19 Pneumonia. N Engl J Med, 2020 ; 384: 20-30.

60. Salvarani C, Dolci G, Massari M, et al. Effect of Tocilizumab vs Standard Care on Clinical Worsening in Patients Hospitalized With COVID-19 Pneumonia: A Randomized Clinical Trial. JAMA Intern Med, 2021 ; 181: 24–31.

61. Salvati L, Occhipinti M, Gori L, et al. Pulmonary vascular improvement in severe COVID-19 patients treated with tocilizumab. Immunol Lett, 2020 ; 228: 122-128.

62. Somers EC, Eschenauer GA, Troost JP, et al. Tocilizumab for treatment of mechanically ventilated patients with COVID-19. Clin Infect Dis, 2020 ; ciaa954 [Online ahead of print]. Available from: <https://doi.org/10.1093/cid/ciaa954>.

63. Stone JH, Frigault MJ, Serling-Boyd NJ, et al. Efficacy of Tocilizumab in Patients Hospitalized with Covid-19. N Engl J Med, 2020 ; 383: 2333-2344.

64. Taboada M, Rama P, Pita-Romero R, et al. Critically ill COVID-19 patients attended by anesthesiologists in northwestern Spain: A multicenter prospective observational study. Rev Esp Anestesiol Reanim, 2021 ; 68: 10-20.

65. Tsai A, Diawara O, Nahass RG, et al. Impact of tocilizumab administration on mortality in severe COVID-19. Sci Rep ; 10: 19131.

66. Veiga VC, Prats JAGG, Farias DLC, et al. Effect of tocilizumab on clinical outcomes at 15 days in patients with severe or critical coronavirus disease 2019: randomised controlled trial. BMJ, 2021 ; 372: 84.

67. Vena A, Giacobbe DR, Di Biagio A, et al. Clinical characteristics, management and in-hospital mortality of patients with coronavirus disease 2019 in Genoa, Italy. Clinical Microbiology and Infection, 2020 ; 26: 1537–1544.

68. Wadud N, Ahmed N, Shergill M, et al. Improved survival outcome in SARs-CoV-2 (COVID-19) acute respiratory distress syndrome patients with tocilizumab administration. Chest, 2020 ; 158: A696-A697.

69. Zantah M, Dominguez-Castillo E, Gangemi, AJ, et al. Anakinra and Intravenous IgG versus Tocilizumab in the Treatment of COVID-19 Pneumonia. MedRxiv 2020.2009.2011.20192401 [Preprint]. Sep 13, 2020 [cited 2021 Jan 20]. Availiable from: https://doi.org/10.1101/2020.09.11.20192401.

70. Zhao H, Zhu Q, Zhang C, et al. Tocilizumab combined with favipiravir in the treatment of COVID-19: a multicenter trial in a small sample size. Biomed Pharmacother, 2021 ; 133: 110825.

71. Galván-Román JM, Rodríguez-García SC, Roy-Vallejo E, et al. IL-6 serum levels predict severity and response to tocilizumab in COVID-19: An observational study. J Allergy Clin Immunol, 2021 ; 147: 72-80.

**Appendix: PRISMA statement[5]**

5. Liberati A, Altman DG, Tetzlaff J, et al. The PRISMA statement for reporting systematic reviews and meta-analyses of studies that evaluate health care interventions: explanation and elaboration. J Clin Epidemiol **2009**; 62(10): e1-34.
